# Hibernation slows epigenetic ageing in yellow-bellied marmots

**DOI:** 10.1038/s41559-022-01679-1

**Published:** 2022-03-07

**Authors:** Gabriela M. Pinho, Julien G. A. Martin, Colin Farrell, Amin Haghani, Joseph A. Zoller, Joshua Zhang, Sagi Snir, Matteo Pellegrini, Robert K. Wayne, Daniel T. Blumstein, Steve Horvath

**Affiliations:** 1 Department of Ecology and Evolutionary Biology, University of California, Los Angeles, CA USA; 2 CAPES Foundation, Ministry of Education of Brazil, Brasília, Brazil; 3 Department of Biology, University of Ottawa, Ottawa, Ontario Canada; 4 Department of Molecular, Cell and Developmental Biology, University of California, Los Angeles, CA USA; 5 Human Genetics, David Geffen School of Medicine, University of California, Los Angeles, CA USA; 6 Biostatistics, Fielding School of Public Health, University of California, Los Angeles, Los Angeles, CA USA; 7 Department of Evolutionary & Environmental Biology, Institute of Evolution, University of Haifa, Haifa, Israel; 8 Rocky Mountain Biological Laboratory, Crested Butte, CO USA

**Keywords:** Epigenetics, Ecological genetics

## Abstract

Species that hibernate generally live longer than would be expected based solely on their body size. Hibernation is characterized by long periods of metabolic suppression (torpor) interspersed by short periods of increased metabolism (arousal). The torpor–arousal cycles occur multiple times during hibernation, and it has been suggested that processes controlling the transition between torpor and arousal states cause ageing suppression. Metabolic rate is also a known correlate of longevity; we thus proposed the ‘hibernation–ageing hypothesis’ whereby ageing is suspended during hibernation. We tested this hypothesis in a well-studied population of yellow-bellied marmots (*Marmota flaviventer*), which spend 7–8 months per year hibernating. We used two approaches to estimate epigenetic age: the epigenetic clock and the epigenetic pacemaker. Variation in epigenetic age of 149 samples collected throughout the life of 73 females was modelled using generalized additive mixed models (GAMM), where season (cyclic cubic spline) and chronological age (cubic spline) were fixed effects. As expected, the GAMM using epigenetic ages calculated from the epigenetic pacemaker was better able to detect nonlinear patterns in epigenetic ageing over time. We observed a logarithmic curve of epigenetic age with time, where the epigenetic age increased at a higher rate until females reached sexual maturity (two years old). With respect to circannual patterns, the epigenetic age increased during the active season and essentially stalled during the hibernation period. Taken together, our results are consistent with the hibernation–ageing hypothesis and may explain the enhanced longevity in hibernators.

## Abstract (web summary)

Species that hibernate generally have longer lifespans than expected based on their body size. The authors show epigenetic ageing patterns from a natural population of hibernating yellow-bellied marmots consistent with the hypothesis that ageing is suspended during hibernation.

## Main

Ageing is a poorly understood natural phenomenon, characterized by an age-progressive decline in intrinsic physiological function^1,2^. The high variation in disease and functional impairment risk among same-age individuals shows that biological age is uncoupled from chronological age^3–5^. Some individuals age at faster rates than others, and little is known about the causes of this inter-individual variance in biological ageing rates^6,7^. To this end, researchers have been attempting to develop biomarkers of ageing^4,8^. Age estimators based on DNA methylation, also known as epigenetic clocks (ECs), are arguably the most accurate molecular estimators of age^3,9–12^. An EC is usually defined as a penalized regression, where chronological age is regressed on methylation levels of individual cytosines^13^. The EC has been successfully used to study human ageing and is becoming increasingly used to study ageing in other species^14–20^.

Age-adjusted estimates of epigenetic age (or epigenetic age acceleration) are associated with a host of age-related conditions and stress factors, such as cumulative lifetime stress^21^, smoking habits^22,23^, all-cause mortality^24–28^ and age-related conditions/diseases^13,26,29,30^. These associations suggest that epigenetic age is an indicator of biological age^5,31^. In fact, measures of epigenetic ageing rates are associated with longevity at the individual level as well as across mammalian species^6,17^. Several studies have presented evidence that long-lived species age more slowly at an epigenetic level^19,20,31–33^. The link between epigenetic ageing and biological ageing is further reinforced by the observation that treatments known to increase lifespan significantly slow the EC^15,17^.

Longevity is related to body size, but some species have longer lifespans than expected based on their body size^34,35^. Many of these long-lived species are able to engage in bouts of torpor^36,37^. Torpor may slow ageing because it is characterized by a dramatic decrease in gene expression and metabolic rate^38–46^. During hibernation, torpor bouts are interspersed by short periods of euthermy (<24 h), when gene expression occurs and metabolism fully recovers^41,47^. Some of the physiological stresses from the cyclic transition between deep torpor and euthermy are similar to the ones experienced by the ageing body (for example, oxidative stress), and promote responses in cellular signalling pathways that are essential for both longevity and torpor survival^36,46^. Thus, the cellular and molecular stress responses associated with torpor–arousal cycles and the long hypometabolic periods may suppress aging^36,46^.

We test the hypothesis that ageing is reduced during hibernation, which we refer to as the ‘hibernation–ageing hypothesis’. Specifically, a species that engages in torpor may periodically ‘suspend’ ageing, as has been previously suggested^36,48,49^. With this rationale, we predict that epigenetic ageing is faster during the active season and slower during hibernation. We test this prediction in yellow-bellied marmots (*Marmota flaviventer*), which spend 7–8 months per year hibernating^50^. Torpor bouts represent 88.6% of the yellow-bellied marmot hibernation period, resulting in an average energy saving of 83.3% when compared with the energetic expenditure of a euthermic adult^50,51^.

## Results

**Fig. 1:**
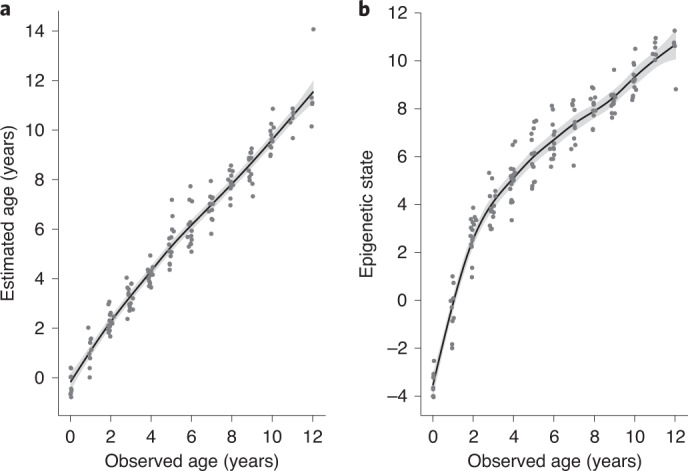
Epigenetic ageing models for a wild population of yellow-bellied marmots. The epigenetic clock (**a**) and the epigenetic pacemaker (**b**). Points represent samples from individuals of known age at the sampling moment (observed age) and the *y* axis represents the epigenetic age calculated by each model. Trend lines were developed by fitting cubic splines. Buffers illustrate the 95% confidence intervals.

We estimated epigenetic age from 149 blood samples collected throughout the life of 73 females using two approaches: the epigenetic clock (EC) and the epigenetic pacemaker (EPM). The epigenetic ageing models developed with the EC and the EPM approaches were both highly accurate (Fig. 1), showing high correlations between epigenetic and chronological age (Pearson correlation coefficient *r* = 0.98 and 0.92, respectively). The EC model had a median absolute error of 0.381 years and a median relative absolute error of 0.084 years, while the EPM model had a median absolute error of 0.899 years and a median relative absolute error of 0.145 years.

Because the effects of season on epigenetic ageing may be nonlinear and potentially cyclical, we used generalized additive mixed models (GAMMs) to test the hibernation–ageing hypothesis. The GAMM that best explained epigenetic ageing calculated from both EPM and EC (Akaike information criterion (ΔAIC) > 2) had the fixed effects of chronological age (cubic spline function) and day of year (cyclic cubic spline function), and random effect of individual identity. Day of year ranged from 1 to 365, with 1 representing 1 May and 365 representing 30 April. Details on the model selection results can be found in Supplementary Table 4. For the EC epigenetic age, this model explained 96.5% of the variation (adjusted *R*^2^) and had a residual variance of 0.362. The random effect of individual identity had no intercept variance. The age spline was significant (effective degrees of freedom (e.d.f.) = 3.19, *F* = 1,278, *P* < 0.0001) and the cyclic spline for day of year was not significant (*P* = 1). Details of this model are described in Supplementary Table 5.

**Fig. 2:**
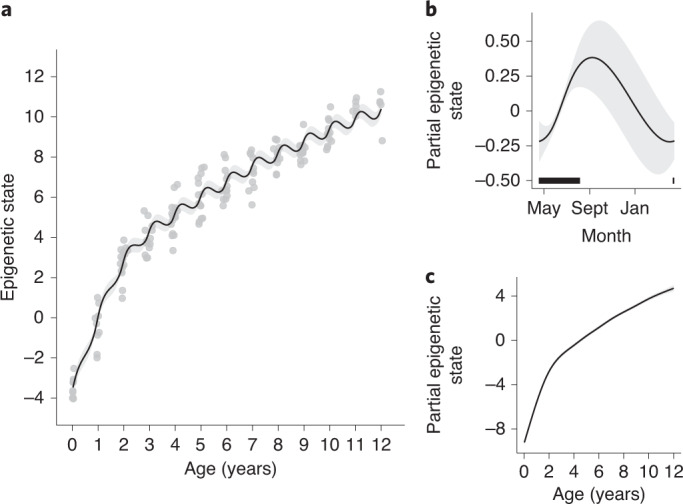
Visualization of the GAMM with epigenetic states generated from the EPM model using CpG sites highly correlated to chronological age (absolute *r* > 0.7). **a**, Changes in the epigenetic state (or epigenetic age) as individuals age. Points are actual data, while lines are the predictions from the model. **b**, Predictions generated with the partial effect of date of year (cyclic cubic smoother spline) on epigenetic state. The black horizontal bar represents when samples were collected and most of the marmot active season. **c**, Predictions generated with the partial effect of chronological age (cubic smoother spline) on epigenetic state. Buffers illustrate the 95% confidence intervals.

The GAMM fitted to the EPM epigenetic state data explained 95.5% of the variation and had a residual variance of 0.294. Both smooth terms significantly influenced marmot epigenetic state (*P* < 0.005; Table 1). The effects of chronological age and day of year result in a particular pattern of epigenetic state change (Fig. 2a), where epigenetic state changes more rapidly during the marmots’ active season. The partial effect of day of year on epigenetic state shows an increase in epigenetic state during the active season and suggests a reversal of such changes during hibernation (Fig. 2b). Moreover, the rate of epigenetic state increase is the highest in the mid-point of the active season. The partial effect of chronological age shows that the epigenetic state increases at a higher rate until females reach two years old, followed by a deceleration as individuals become older (Fig. 2c).

**Table 1:** Output from the GAMM using epigenetic states (or epigenetic ages) estimated from EPM models as dependent variable

	Estimate	Standard error	*t* value	*P* value	e.d.f.	Reference d.f.	*F*	Random effect (animal ID)
**Intercept**	5.53	0.09	63.22	<0.0001				0.329
**Residual**								0.294
**Smooth term: age**^a^ **(cubic spline)**				<0.0001	7.15	7.15	339.20	
**Smooth term: date**^b^ **(cyclic spline)**				0.002	1.39	8.00	3.27	

^a^Individual chronological age in years calculated from the first time an individual emerged from their mother’s burrow to the date they were captured.

^b^Day of the year (values varied from 1 to 365, with 1 representing 1 May and 365 representing 30 April).

*t* value is derived from the *t* statistic used in the GAMM to test for the significance of linear terms in the model.

**Fig. 3:**
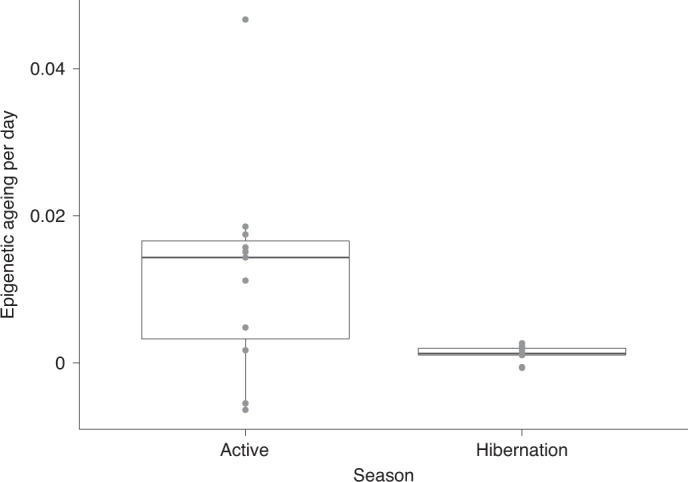
Epigenetic ageing rate during the active and hibernation seasons of 11 yellow-bellied marmots with samples collected in consecutive years. Epigenetic ageing rates were calculated using the epigenetic ages from the EPM model. The points represent the rate of epigenetic ageing calculated for each individual. The individuals with negative rates of epigenetic ageing and the individual with the highest rate during the active season are all old females (details in Supplementary Information). The box includes the rate values in between the first and third quartiles (the 25th and 75th percentiles), the horizontal black line represents the median and the whiskers extend from the box to the largest or lowest value no further than 1.5 times the distance between the first and third quartiles.

To complement these results, we subsampled our data to the 11 individuals trapped in consecutive years, with a minimum of 2 samples in one year and 1 sample in the other year. From this subset, we estimated the rate of EPM epigenetic ageing per individual during the active and hibernation seasons. The measures of epigenetic ageing per day during the active season were significantly higher than during hibernation (Fig. 3, *χ*^2^ = 5.132, degrees of freedom (d.f.) = 1, *P* = 0.024). We observed similar trends for the average change in methylation levels across all 31,388 CpG sites from the mammalian array, suggesting that seasons affect methylation levels at the epigenome level and may not be restricted to age-related sites (Supplementary Information). In this regard, hibernation may influence not only epigenetic ageing but also other biological processes.

### Simulations

We performed two simulation approaches to estimate the type-1 error and the power to detect a hibernation–ageing effect in our GAMMs given the limitations of our sample collection. Specifically, samples could only be collected during the active season, instead of throughout the year. Our earliest sample was collected on 27 April and the latest on 20 August. From the 1,000 GAMMs fitted to data simulated with a seasonal effect, 75.9% found a significant effect of seasons, indicating high power to detect a seasonal effect given the simulated parameters and our data structure. From the 1,000 GAMMs fitted to data simulated with no seasonal effect, 6.2% had a significant season effect, indicating a slightly higher type-1 error than expected (5%). Based on this result from the simulations with no seasonal effect, we calculated a new critical value for the probability that respects the 5% type-1 error rate by estimating the 0.05 quantile of the *P*-value distribution from a null model. The 0.05 quantile was 0.0399, which can be taken as the critical value with which to estimate the significance of a seasonal effect. In our second approach, we simulated 1,000 datasets with permuted (randomly assigned) days of the year and observed a significant seasonal effect for 6.7% of the GAMMs. The adjusted *P*-value threshold based on this result was 0.0416. The *P* value for seasonal effects on the marmot data is 0.002 (Table 1) and therefore is considered significant. From both approaches, we concluded that our results were neither driven by our sampling nor by statistical artefact.

### Age-related CpGs

**Fig. 4:**
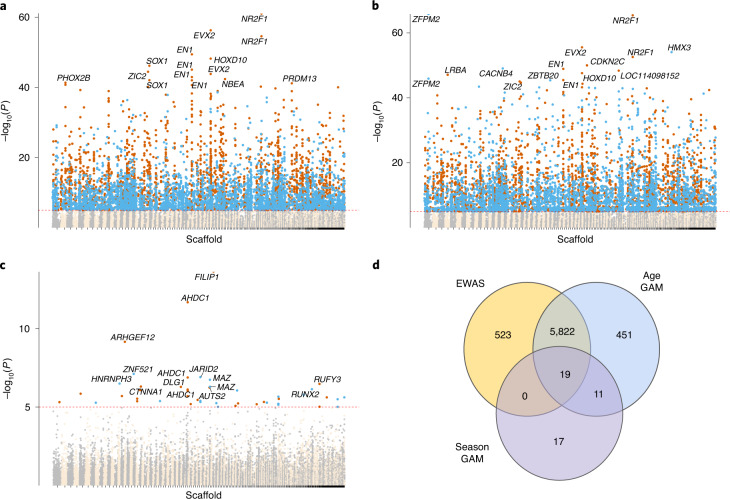
Associations of CpG sites with chronological age and seasons (day of the year) in blood of yellow-bellied marmots. **a**, The *y* axis reports log transformed *P* values for the EWAS of chronological age. **b**,**c**, The *y* axis reports log transformed *P* values for two fixed effects of the GAMs of individual cytosines (dependent variable) for chronological age (**b**; cubic spline function; age GAM) and day of year (**c**; cyclic cubic spline function; season GAM). The CpG sites’ coordinates were estimated based on the alignment of mammalian array probes to yellow-bellied marmot genome assembly. For some of the most significant CpGs, the symbols of proximal genes are provided. The direction of associations with chronological age is highlighted for the significant sites, with orange for hypermethylated and blue for hypomethylated sites; the red dashed lines represent the significance threshold (*P* < 10^−5^). Note that the season effect is cyclical, and we show the direction of association with chronological age for the active season. **d**, Venn diagram showing the overlap of significant (*P* < 10^−^^5^) CpG sites between EWAS and the GAMs. The effects of age and season in the GAMs are represented separately.

In the epigenome-wide association studies (EWAS) of chronological age, the methylation level of 6,364 CpGs was significantly (*P* < 10^−5^) associated with chronological age (Supplementary Table 6). In the generalized additive models (GAMs) per site (Supplementary Table 7), the age effect (cubic smoother spline) was significant in 6,303 sites, which largely overlapped with EWAS of age (Fig. 4d). Significance thresholds were set to 1 × 10^−5^ due to a compromise between the Bonferroni correction and the Benjamini–Hochberg false-detection rate (FDR). The Bonferroni threshold from our data was 1.6e^−06^ and selected 5,317 sites from the GAM (age effect) and 5,440 from the EWAS. The Benjamini–Hochberg FDR is a less conservative FDR correction and selected 14,117 sites from the GAM (age effect) and 13,575 from the EWAS. The threshold used is strict particularly because methylation levels are highly correlated among CpGs, and thus the analyses per site are not independent. Haghani et al.^52^ identified 55 co-methylation modules in the mammalian array, which would lead to a threshold of *P* < 0.001 with a Bonferroni correction.

**Extended Data Fig. 1:**
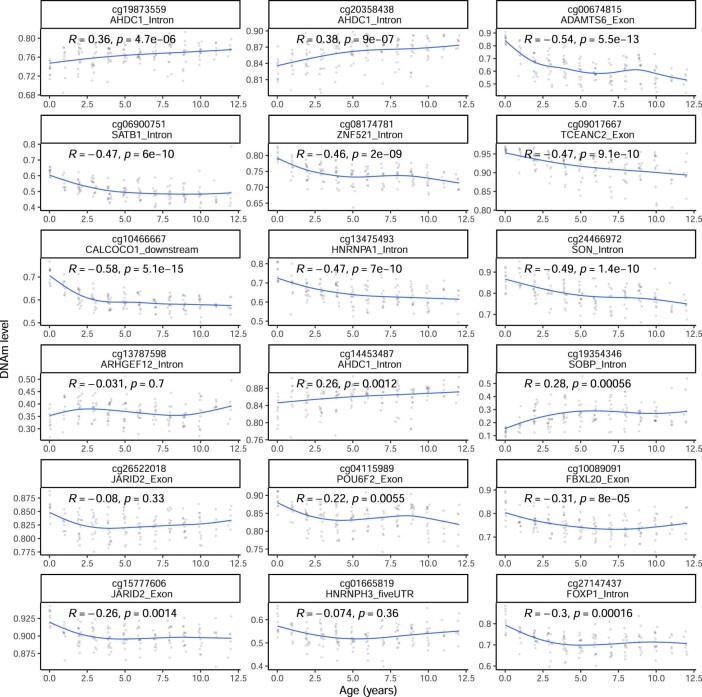
Examples of CpG sites where methylation levels are non-linearly related with chronological age. Each plot includes the site identification in the Mammalian array and its location relative to the closest transcriptional start site. The CpG sites coordinates were estimated based on the alignment of Mammalian array probes to yellow-bellied marmot (Marmota flaviventer) genome assembly. Trend lines represent the GAM smooth function. R and p-values are based on the Pearson correlation of the CpGs’ methylation level and age in marmots.

From the 5,841 sites that overlapped between the EWAS and the age effect (GAMs), 66% (3,827 sites) had e.d.f. values larger than 2 for the age effect in the GAMs. The e.d.f. measures the complexity of the curve, and these results suggest that many CpG sites have a nonlinear relationship with chronological age (examples in Extended Data Fig. 1). Top age-related CpGs in both EWAS and GAMs were located on *NR2F1* and *EVX2* downstream regions (Fig. 4a,b). The promoters of *EN1* and *HOXD10* were also hypermethylated with age. Age-related sites uniquely identified by GAMs were proximal to *FAM172A* intron (hypermethylated), and hypomethylated in both the *CSNK1D* 3′ untranslated region and *HNRNPC* intron.

**Extended Data Fig. 2:**
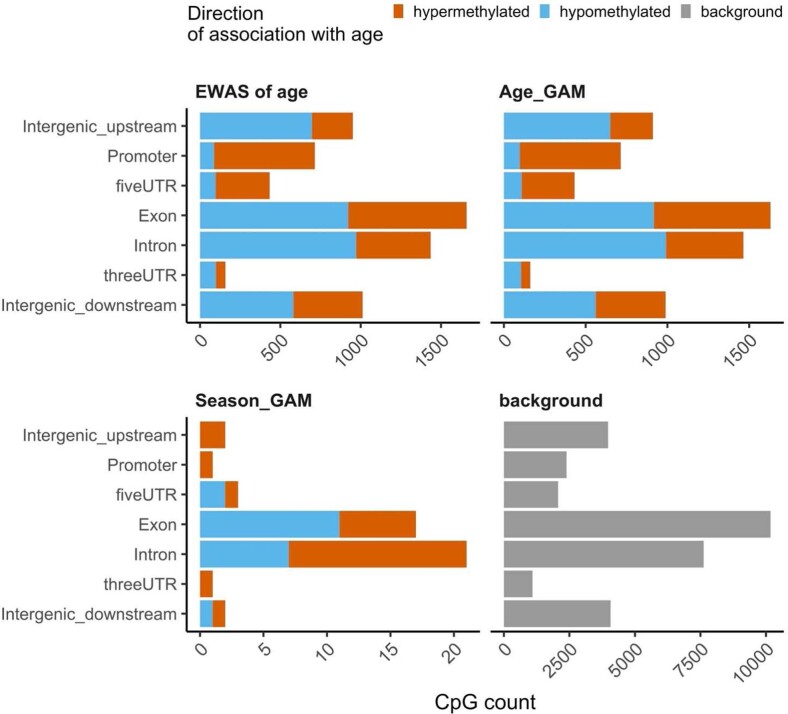
Location of CpG sites relative to the closest transcriptional start site. The CpG sites coordinates were estimated based on the alignment of Mammalian array probes to yellow-bellied marmot (Marmota flaviventer) genome assembly. A,B) CpGs associated with chronological age. C) CpGs associated with seasons (day of the year). D) Location of the Mammalian array probes mapped to the yellow-bellied marmot genome. The direction of associations with chronological age is highlighted (p < 10-5) with orange for hypermethylated and blue for hypomethylated sites. Note that the season effect is cyclical, and we show the direction of association with chronological age for the active season.

**Extended Data Fig. 3:**
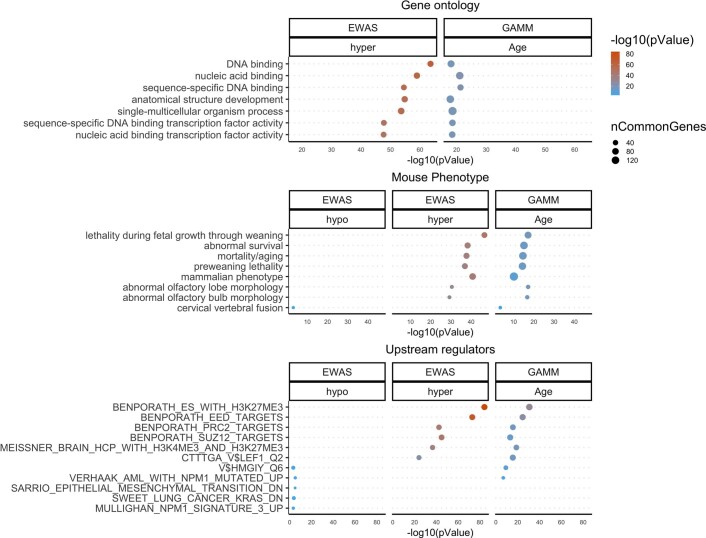
Enrichment analysis of the top ageing-related CpGs in yellow bellied marmots (Marmota flaviventer). The analysis was done using the Genomic Regions Enrichment of Annotations Tool (GREAT). The gene level enrichment was done using human GRCh37 background. The background probes were limited to 19,695 probes that were mapped to the same gene in the marmot genome. The top 3 enriched datasets from each category (Canonical pathways, gene ontology, mouse phenotype, and upstream regulators) were selected and further filtered for significance at p < 10^−3^. Only terms with more than 5 foreground genes and at least 23 background genes were included.

The significant CpGs were located in both genic and intergenic regions relative to transcriptional start sites (Extended Data Fig. 2). Compared with the background, we observed a higher proportion located at promoter regions, where most CpGs were hypermethylated with age (Extended Data Fig. 2). From the 3,914 CpGs used in the enrichment analysis, 396 were located in promoter regions and 87% of those were hypermethylated with age. DNA methylation (DNAm) ageing in marmots was proximal to polycomb repressor complex targets (*PRC2*, *EED*) with *H3K27ME3* marks (Extended Data Fig. 3), which is a consistent observed pattern in all mammals^53^. The enriched pathways were largely associated with development, cell differentiation and homoeostasis. CpG-site annotations and detailed enrichment results are available in the supplementary material (Supplementary Tables 8–10).

### Season-related CpGs

The seasonal effect in the GAMs per site, measured with a cyclic cubic spline function of day of the year, was significantly associated with methylation in 47 CpG sites proximal to 37 genes. Most of the season-related CpGs were also associated with age (Fig. 4d). Some of the most significant CpGs for both season and age effects in the GAMs are proximal to *FILIP1* exon, *ARHGEF12* intron, *ZNF521* intron, *JARID2* exon and *AHDC1* intron (Fig. 4c). The most significant CpGs for the season effect alone are proximal to *AHDC1* intron, *MAZ* exon, *CTNNA1* exon, *AUTS2* intron and *EFNA5* exon (Fig. 4c). The *AHDC1* intron seems to be an interesting region for further exploration because it is proximal to sites solely affected by season, sites only related with age and those influenced by both. Mutations in *AHDC1* are implicated in obstructive sleep apnoea^54^, so this gene may play a role in sleep processes and potentially hibernation. Since the seasonal effect size is smaller and more nonlinear than the age effect (Fig. 2), our power to identify sufficient season CpGs for enrichment analysis was limited by our sample size and no biological function was considered significant.

## Discussion

Acquiring chronological-age data from wildlife is a daunting task, but age data have fundamental applications to behavioural ecology, evolutionary biology and animal conservation^7,55^. Epigenetic ageing models promise to inform age estimates and facilitate ageing research in wild and non-model organisms^14,18,55^. We present new epigenetic ageing models for marmots, an excellent animal model to study hibernation. We applied a validated platform for measuring methylation levels (mammalian methylation array^56^) to a unique collection of tissues—blood samples from known-age, free-living animals—to investigate how ageing is affected by active-hibernation cycles.

The EPM results showed a rapid change in epigenetic age until marmots reached two years old, their age of sexual maturity^57,58^. After reaching adulthood, change in epigenetic age was more linear and slower, which is similar to the pattern observed in humans older than 20 years^59^. The pattern observed in marmot epigenetic ageing is consistent with the notion that methylation remodelling is associated with key physiological milestones^33^. A logarithmic relationship between methylation change rate and chronological age may be a shared trait in mammals, and such a relationship has been described for multiple human tissues^9,59,60^ and some species, including dogs^33^ and mice^15^.

With regard to active and hibernation seasons, the EC model was unable to capture seasonal effects because it uses a penalized regression to relate the dependent variable (chronological age) to cytosines. The EPM is better equipped to detect nonlinear and potentially cyclic patterns because it estimates the epigenetic state by minimizing the error between estimated and measured methylation levels^59,61^, which allows for a nonlinear relationship of methylation levels with chronological age. Since ageing rate is not constant throughout an individual’s lifespan^62,63^, the EPM is possibly more influenced by factors associated with biological aging^61^.

According to both analyses that used EPM-estimated epigenetic age, biological ageing slows during hibernation. Specifically, the clear delay in epigenetic state changes during hibernation supports our hibernation–ageing hypothesis. Interestingly, this hypothesis does not seem to hold for individuals before sexual maturity. Even though we observed a non-significant interaction between chronological age and day of year, our model predictions indicated a weaker deceleration in ageing during hibernation for individuals in their first and second years of life (Fig. 2a). Compared with adults, young marmots may enter hibernation weeks later^64–66^, spend less time torpid during hibernation and have higher daily mass loss in deep torpor^50^. Indeed, thermoregulatory support from adults increases overwinter survival of young alpine marmots^67–69^. Thus, a weaker effect of slowed ageing during hibernation in younger animals may be explained by their later hibernation start date in addition to an overall higher metabolic rate during hibernation.

The seasonal trends observed in marmot ageing probably occur in other species because molecular and physiological changes during hibernation are similar among mammals^36,41,47,70^. Some indications that hibernation slows ageing exist: Turkish hamsters (*Mesocricetus brandti*) that spent more time hibernating lived longer^71^; black bears with shorter hibernation length had higher telomere attrition^72^ and Djungarian hamsters (*Phodopus sungorus*) frequently using daily torpor had longer relative telomere length (RTL)^49^. RTL was shorter in edible^48^ and garden^73^ dormice (*Glis glis* and *Eliomys quercinus*) that spent more time euthermic during hibernation. However, RTL measured across the active season was elongated in adult edible dormice^48^ and did not change in garden dormice^73^, which is conflicting with the assumption that individuals would age when active. The relationship of RTL with chronological age is not always negative^74–76^, and RTL may be more associated with cell senescence than cell aging^77,78^.

Some of the physiological stresses experienced by individuals during hibernation are similar to those observed with ageing, and therefore the molecular and physiological responses required for an individual to successfully hibernate may prevent aging^36,46^. Additionally, hibernation combines conditions known to promote longevity^36,46,79^, such as food deprivation (calorie restriction^80–82^), low body temperature^79,83–85^ and reduced metabolic rates^46^. Conceivably, these factors may also be associated with the slower marmot ageing observed in the beginning and end of their active season (Fig. 2b). Marmots in early spring and late fall have limited calorie intake^66,86^, reduced overall activity^86–88^ and lower metabolic rate^89^ than during summer. This variation in epigenetic ageing rate within the active season may occur in other mammals. For instance, free-living arctic ground squirrels begin dropping body temperature 45 days before hibernation^90^, 13-lined ground squirrels drop food consumption by 55% before hibernation^91^ and some species exhibit short and shallow torpor bouts before and after hibernation^92^.

DNAm ageing in marmots was related to genes involved in several developmental and differentiation processes, as has been seen in other mammals^17,19,55,93^. This common enrichment across mammals implies an evolutionary conservation in the biological processes underpinning ageing. This inference has been further reinforced by a recent study developing ECs capable of accurately predicting chronological age in distantly related species and, in theory, in any mammalian species^53^. These ‘universal clocks’ for eutherians can be used in any tissue sample and are developed from CpG sites located in conserved genomic regions across mammals^56^.

Seasonally dynamic methylation levels were identified in 47 CpG sites. Although few CpGs were identified in our analysis per site, the effect of season was detected by the EPM algorithm, which represents methylation changes in all sites correlated (absolute *r* > 0.7) with chronological age^59,61^. Thus, seasonality probably influences many more CpGs in common with ageing than we were able to detect. Nevertheless, many of the top season-related sites were proximal to genes with circannual patterns in other species. For instance, *AUTS2* is differentially expressed across seasons and within hibernation in brown adipose tissue of 13-lined ground squirrels^94^ and its proximal CpGs are differentially methylated in blood and liver throughout the reproductive season of great tits^95^. *JARID2* is differentially expressed within hibernation in the cerebral cortex of 13-lined ground squirrels^96^ and seasonally expressed in human peripheral blood mononuclear cells^97^. *RUFY3* is differentially expressed between active and hyperphagia phases in the subcutaneous adipose tissue of grizzly bears^98^ and is close to season-related CpGs in great tits^99^. Methylation levels of sites close to *FILIP1*, *AHDC1*, *ARHGEF12*, *ZNF521*, *CTNNA1* and *AUTS2* vary seasonally in great tits^99^. *ARHGEF12* is also upregulated in songbirds exhibiting migratory behaviour^100^. The expression of these genes may thus be of some importance to species with seasonal behaviour, including in hibernating and non-hibernating species.

In sum, we observed a substantial deceleration in epigenetic ageing during hibernation. While hibernation may increase longevity by protecting individuals from predators and diseases^37^, we suggest that the biological processes involved in hibernation are important contributors to the long lifespan seen in most hibernators. A mechanistic understanding of the anti-ageing properties of hibernation will be further advanced by the exploration of the intra- and inter-specific variation in torpor use (for example, length and frequency of torpor–arousal cycles^50,101–104^) and the many survival strategies associated with metabolic rate depression (for example, anoxia and freeze tolerance^47,105,106^). Since metabolic depression is reached through similar molecular and biochemical patterns across the animal kingdom^107^, ageing may be moulded by these life history traits by similar evolutionary pathways. Longevity is a key component of individual fitness, therefore improved understanding of the pathways linking hibernation and ageing has multiple potential applications, including for species unable to enter torpor. In addition to the potential biomedical^36,46^ and space exploration^108^ implications, studying torpor in multiple species can provide new insights into the mechanisms of ageing and the reasons for variation in biological ageing rates among individuals and species.

## Methods

All samples were collected as part of a long-term study of a free-living population of yellow-bellied marmots in the Gunnison National Forest, Colorado (USA), where marmots were captured and blood samples collected biweekly during their active season (May to August^109^). Data and samples were collected under the University of California, Los Angeles (UCLA) Institutional Animal Care and Use protocol (2001-191-01, renewed annually) and with permission from Colorado Parks and Wildlife (TR917, renewed annually). Individuals were monitored throughout their lives, and chronological age was calculated based on the date at which juveniles first emerged from their natal burrows. We only used female samples because precise age for most adult males is unavailable since males are typically immigrants born elsewhere^57,110^. We selected 160 blood samples from 78 females with varying ages.

Genomic DNA was extracted with Qiagen DNeasy blood and tissue kit and quantified with Qubit. DNAm profiling was performed with the custom Illumina chip HorvathMammalMethylChip40 (ref. ^56^). This array, referred to as mammalian methylation array, profiles 36,000 CpG sites in conserved genomic regions across mammals. From all probes, 31,388 mapped uniquely to CpG sites (and their respective flanking regions) in the yellow-bellied marmot assembly (GenBank assembly accession: GCA_003676075.2). We used the SeSaMe normalization method to estimate methylation levels (*β* values) for each CpG site^111^.

We used two different unsupervised hierarchical clustering procedures to identify technical outliers. The first clustering procedure was based on imputed single nucleotide polymorphisms (SNPs). Toward this end, we used MethylToSNP v.0.99.0 (ref. ^112^) to identify CpG sites that corresponded to SNPs. The SNP data were used for unsupervised hierarchical clustering based on Euclidean distances. Branches (clusters) of the cluster tree corresponded to multiple samples from the same animal. This allowed us to identify a small plate map error probably caused by human pipetting error. To err on the side of caution, we removed putative plate map errors from the dataset. Second, we carried out average linkage hierarchical clustering based on the inter-array correlation to identify technical outliers due to an insufficient amount of DNA. The DNAm profiling from 149 samples passed quality control. These samples were collected from 73 females (1 to 8 samples per individual) with ages varying from 0.01 to 12.04 years.

Two model approaches were used to study epigenetic ageing in marmots: the epigenetic clock^9,10^ and the epigenetic pacemaker^59–61,113^. Both models are described below.

### Epigenetic clock

Under the EC, a linear correlation with age is determined by attempting to fit a single coefficient to each CpG site. We fitted a generalized linear model with elastic-net penalization^114^ to the chronological-age and *β*-value datasets using the glmnet v.4.0-2 package in R^115^. *α* was set to 0.5, which assigns ridge and lasso penalties with the same weight. The elastic-net penalization limits the impact of collinearity and shrinks irrelevant coefficients to zero. This method estimates coefficients that minimize the mean squared error between chronological and predicted ages and performs an automatic selection of CpG sites for age prediction. We applied a 10-fold cross validation to select the model with lowest error based on the training set. Predicted ages were scored for samples not included in the training set of the model. In this respect, the predicted age was estimated for groups of ~14 samples, resulting in 11 EC models using a total of 360 sites (Supplementary Table 1). We also report the coefficient per site, intercept and *λ* (the regularization parameter value with minimum mean cross-validated error) from the EC using all data as the training set (Supplementary Table 2).

### Epigenetic pacemaker

While ECs are used to estimate the age of a sample based on weighted sums of methylation values, the EPM models the dynamics of methylation across the genome. To accomplish this, it models each individual CpG site as a linear function of an underlying epigenetic state of an individual. This epigenetic state changes with time in a nonlinear fashion and can therefore be used to identify periods with variable rates of methylation changes throughout lifespan. The EPM assumes that the relative increase/decrease in rate of methylation levels among sites remains constant, but the absolute rates can be modified when rates at all sites change in synchrony^59,61,113^. The optimum values of methylation change rate and initial methylation level per site, as well as the epigenetic state per sample, are calculated through iterations implemented in a fast conditional expectation maximization algorithm^116^ to minimize the residual sum of squares error between known and estimated methylation levels (*β* values). Thus, the epigenetic state is an estimate of age that, given the methylation rates and initial methylation levels for each site, minimizes the differences between known and estimated methylation levels in a specific sample for all sites included in the model. We selected sites to use in the EPM v.0.0.3 based on the absolute Pearson correlation coefficients (*r*) between chronological age and methylation levels per site^59,61^. All sites with absolute *r* > 0.7 were included, which resulted in 309 sites. A 10-fold cross validation was used to estimate epigenetic states. We report the rate and intercept values per site from the EPM using all data as the training set (Supplementary Table 3).

### Hibernation–ageing hypothesis

We performed model selection based on the Akaike information criterion for GAMMs fitted to the EPM- or the EC-estimated epigenetic-age datasets. For each epigenetic-age dataset, eight GAMMs with different fixed effects were compared: (1) chronological age and day of year; (2) the interaction between chronological age and day of year; (3) cubic spline function for chronological age and day of year; (4) chronological age and a cubic spline function for day of year; (5) chronological age and a cyclic cubic spline function for day of year; (6) cubic spline function for chronological age and a cubic spline function for day of year; (7) cubic spline function for chronological age and a cyclic cubic spline function for day of year and (8) the interaction (using tensor product interaction) between a cubic spline function for chronological age and a cyclic cubic spline function for day of year. Individual identity was added as a random effect in all models. Day of year ranged from 1 to 365, with 1 representing 1 May and 365 representing 30 April.

**Extended Data Fig. 4:**
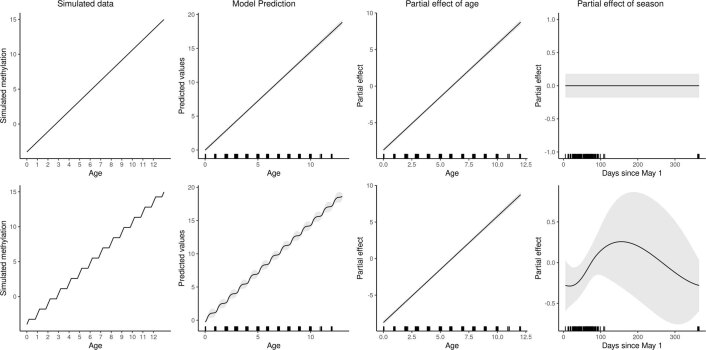
Visualization of the simulated patterns of epigenetic ageing. We simulated two traits: a linear trait (first row) that increases linearly with age independently of the season; and a seasonal trait (second row) that increases during the active season but not during the hibernation. For each trait we show the raw simulated data, the prediction from the GAMM fitted to the simulated data, and the predictions of the partial effects of chronological age and season from the model.

We used two simulation approaches to estimate the type-1 error and the power to detect a hibernation–ageing effect given the limitations of our sample collection. Specifically, blood samples could only be collected during the active season, instead of throughout the year. Our earliest sample was collected on 27 April and the latest on 20 August. In our first approach, we simulated two traits (Extended Data Fig. 4): (1) a trait that increases linearly with age independently of the season and (2) a trait that increases during the summer but not during the winter. The daily rate of increase for the first trait was set at 0.004, to simulate data with a similar range to the observed EPM data. For the second trait, the rate of increase was set to 0 during winter (16 September to 17 April, days 139–352 using 1 May as reference). The simulation assumed that the active season was 150 days long starting on 18 April (day 353) and finishing on 15 Sept (day 138). The rate of increase during the active season was set as 0.0164 (0.004/365 × 150) so that the annual rate of increase was similar between the two simulated traits. Our simulation was parametrized using among-individual and residual variance from the EPM. We performed these simulations using field data (day of sample collection, age in days, birth date and number of samples) and estimated the significance of the seasonal effect with the GAMM that best explained the marmot data (model selection described above). We repeated this procedure 1,000 times for both traits. The proportion of simulations on trait 1 (no seasonal effect) that were significant indicated our type-1 error. The proportion of simulations on trait 2 (seasonal effect) that were significant was an indication of the power to detect this effect. In the second approach, we simulated 1,000 datasets where day of year was randomly shuffled. The type-1 error was estimated as the proportion of simulations with significant seasonal effects in the GAMMs.

We evaluated GAMMs by checking convergence, concurvity between fixed effects and the autocorrelation of deviance residuals. We also checked model fit by plotting response versus fitted values and visually inspected qq plots and histograms of deviance residuals, plots of deviance residuals with fitted values and plots of deviance residuals with explanatory variables. GAMMs were fitted and checked using the gamm4 R package v.0.2-6 (ref. ^117^). All analysis and figures were developed in R v.3.6.3 (ref. ^118^) in RStudio v.1.2.5033 (ref. ^119^), python v.3.7.4 (ref. ^120^), Jupyter notebook v.6.0.3 (ref. ^121^), ggplot2 v.3.3.0.9 (ref. ^122^) and ggpubr v.0.2.5 (ref. ^123^).

To complement the results from the GAMMs, we subsampled our data for the individuals with samples collected in consecutive years. We further restricted this subset for the cases where two or more samples were collected in one year (that is, active season intervals) and at least one other sample in the other year (that is, hibernation intervals). To measure the active EPM epigenetic ageing rate, we subtracted the EPM epigenetic age of the earliest sample from the latest sample in the active season, and divided by the number of days in between the sampling dates. To measure hibernation EPM epigenetic ageing rate, we subtracted the EPM epigenetic age of the latest sample before hibernation from the EPM epigenetic age from the first sample collected after hibernation, divided by the number of days between the collection events. The significance of the difference between the epigenetic ageing rate during the active and hibernation seasons was estimated with Kruskal–Wallis rank sum test.

### Influence of chronological age and seasons on methylation levels per CpG site

We performed additional analyses to identify which CpG sites were associated with age and seasonality. We fitted a GAM per CpG site with the mgcv R package v.1.8 (ref. ^124^), where methylation level was the dependent variable. The independent variables were a cubic spline function for chronological age and a cyclic cubic spline function for day of year.

Since EWAS have been more commonly used to identify CpG sites related to chronological age, we performed a linear regression per CpG site with lmerTest v.3.1-3 (ref. ^125^). Each model had methylation level as dependent variable and chronological age as independent variable.

### CpG-site enrichment analysis

Gene enrichment was performed with the Genomic Regions Enrichment of Annotations Tool v.3.0.0 (GREAT hypergeometric test^126^). GREAT analyses the potential cis-regulatory role of the non-coding regions with CpG sites of interest, and identifies which pathways are overrepresented in the data. To associate CpGs with genes, we used the ‘Basal plus extension’ association with a maximum window distance between the CpG and the genes of 50 kb. GREAT tests the observed distribution of CpG neighbouring genes against the expected number of sites associated with each pathway due to their representation in the mammalian array (background set). Since GREAT requires a high-quality annotation, we used the respective locations of the marmot sites on the human assembly (GRCh37), and therefore only used sites mapped to conserved genes between marmots and humans. The background set included 19,695 sites. Two datasets were analysed: sites associated with chronological age and with day of year. The alignment and annotation methods are described in the mammalian methylation array method paper^56^.

### Reporting Summary

Further information on research design is available in the Nature Research Reporting Summary linked to this article.

## Supplementary information

Supplementary Information Additional analysis evaluating the effect of the CpGs inputted in the EPM on GAMM results and testing the hibernation–ageing hypothesis with samples collected from same individuals across consecutive years.

Reporting Summary.

Peer Review Information.

Supplementary Tables 1–10.

Supplementary Data 1 Information for each sample for: OriginalOrderInBatch (order in which each sample was analysed), Basename (sample identification), Age (age in years of individuals at sampling time), AnimalID (individual identification), Trap_jday (Julian date of sample collection), Epigenetic_state (epigenetic age measured with the EPM), EC_predicted (epigenetic age measured with the EC) and pup_emerjdate (date of first burrow emergence after individuals were born).

## Data Availability

Epigenetic data are deposited in the Gene Expression Omnibus GSE174544 and available at 10.17605/OSF.IO/E42ZV^127^.

## Code availability

Code is available at 10.17605/OSF.IO/E42ZV^127^.

## References

[1] Flatt T (2012). A new definition of aging?. Front. Genet..

[2] Berdasco M, Esteller M (2012). Hot topics in epigenetic mechanisms of aging: 2011. Aging Cell.

[3] Jylhävä J, Pedersen NL, Hägg S (2017). Biological age predictors. EBioMedicine.

[4] Wagner KH, Cameron-Smith D, Wessner B, Franzke B (2016). Biomarkers of aging: from function to molecular biology. Nutrients.

[5] Field AE (2018). DNA methylation clocks in aging: categories, causes, and consequences. Mol. Cell.

[6] Horvath S (2015). Decreased epigenetic age of PBMCs from Italian semi-supercentenarians and their offspring. Aging.

[7] Nussey DH, Froy H, Lemaitre JF, Gaillard JM, Austad SN (2013). Senescence in natural populations of animals: widespread evidence and its implications for bio-gerontology. Ageing Res. Rev..

[8] Johnson TE (2006). Recent results: biomarkers of aging. Exp. Gerontol..

[9] Horvath S (2013). DNA methylation age of human tissues and cell types. Genome Biol..

[10] Hannum G (2013). Genome-wide methylation profiles reveal quantitative views of human aging rates. Mol. Cell.

[11] Unnikrishnan A (2019). The role of DNA methylation in epigenetics of aging. Pharmacol. Ther..

[12] Bocklandt S (2011). Epigenetic predictor of age. PLoS ONE.

[13] Horvath S, Raj K (2018). DNA methylation-based biomarkers and the epigenetic clock theory of ageing. Nat. Rev. Genet..

[14] Polanowski AM, Robbins J, Chandler D, Jarman SN (2014). Epigenetic estimation of age in humpback whales. Mol. Ecol. Resour..

[15] Petkovich DA (2017). Using DNA methylation profiling to evaluate biological age and longevity interventions. Cell Metab..

[16] Stubbs TM (2017). Multi-tissue DNA methylation age predictor in mouse. Genome Biol..

[17] Wang T (2017). Epigenetic aging signatures in mice livers are slowed by dwarfism, calorie restriction and rapamycin treatment. Genome Biol..

[18] Ito G, Yoshimura K, Momoi Y (2017). Analysis of DNA methylation of potential age-related methylation sites in canine peripheral blood leukocytes. J. Vet. Med. Sci..

[19] Thompson MJ, von Holdt B, Horvath S, Pellegrini M (2017). An epigenetic aging clock for dogs and wolves. Aging.

[20] Lowe R (2018). Ageing-associated DNA methylation dynamics are a molecular readout of lifespan variation among mammalian species. Genome Biol..

[21] Zannas AS (2015). Lifetime stress accelerates epigenetic aging in an urban, African American cohort: relevance of glucocorticoid signaling. Genome Biol..

[22] Zaghlool SB (2015). Association of DNA methylation with age, gender, and smoking in an Arab population. Clin. Epigenetics.

[23] Gao X, Zhang Y, Breitling LP, Brenner H (2016). Relationship of tobacco smoking and smoking-related DNA methylation with epigenetic age acceleration. Oncotarget.

[24] Marioni RE (2016). The epigenetic clock and telomere length are independently associated with chronological age and mortality. Int. J. Epidemiol..

[25] Marioni RE (2015). DNA methylation age of blood predicts all-cause mortality in later life. Genome Biol..

[26] Perna L (2016). Epigenetic age acceleration predicts cancer, cardiovascular, and all-cause mortality in a German case cohort. Clin. Epigenetics.

[27] Chen BH (2016). DNA methylation‐based measures of biological age: meta‐analysis predicting time to death. Aging.

[28] Christiansen L (2016). DNA methylation age is associated with mortality in a longitudinal Danish twin study. Aging Cell.

[29] Horvath S, Levine AJ (2015). HIV-1 infection accelerates age according to the epigenetic clock. J. Infect. Dis..

[30] Horvath S (2015). Accelerated epigenetic aging in Down syndrome. Aging Cell.

[31] Parrott BB, Bertucci EM (2019). Epigenetic aging clocks in ecology and evolution. Trends Ecol. Evol..

[32] Wagner W (2017). Epigenetic aging clocks in mice and men. Genome Biol..

[33] Wang T (2020). Quantitative translation of dog-to-human aging by conserved remodeling of the DNA methylome. Cell Syst..

[34] Wilkinson GS, Adams DM (2019). Recurrent evolution of extreme longevity in bats. Biol. Lett..

[35] Austad SN (2009). Comparative biology of aging. J. Gerontol. A.

[36] Wu CW, Storey KB (2016). Life in the cold: links between mammalian hibernation and longevity. Biomol. Concepts.

[37] Turbill C, Bieber C, Ruf T (2011). Hibernation is associated with increased survival and the evolution of slow life histories among mammals. Proc. R. Soc. Lond. B.

[38] Chen Y (2001). Mechanisms for increased levels of phosphorylation of elongation factor-2 during hibernation in ground squirrels. Biochemistry.

[39] Knight JE (2000). mRNA stability and polysome loss in hibernating Arctic ground squirrels (*Spermophilus parryii*). Mol. Cell. Biol..

[40] Yan J, Barnes BM, Kohl F, Marr TG (2008). Modulation of gene expression in hibernating arctic ground squirrels. Physiol. Genomics.

[41] Van Breukelen F, Martin SL (2002). Molecular adaptations in mammalian hibernators: unique adaptations or generalized responses?. J. Appl. Physiol..

[42] Morin P, Storey KB (2006). Evidence for a reduced transcriptional state during hibernation in ground squirrels. Cryobiology.

[43] van Breukelen F, Martin SL (2002). Reversible depression of transcription during hibernation. J. Comp. Physiol. B.

[44] Azzu V, Valencak TG (2017). Energy metabolism and ageing in the mouse: a mini-review. Gerontology.

[45] Schrack JA, Knuth ND, Simonsick EM, Ferrucci L (2014). ‘IDEAL’ aging is associated with lower resting metabolic rate: the Baltimore Longitudinal Study of Aging. J. Am. Geriatr. Soc..

[46] Al-attar R, Storey KB (2020). Suspended in time: molecular responses to hibernation also promote longevity. Exp. Gerontol..

[47] Carey HV, Andrews MT, Martin SL (2003). Mammalian hibernation: cellular and molecular responses to depressed metabolism and low temperature. Physiol. Rev..

[48] Turbill C, Ruf T, Smith S, Bieber C (2013). Seasonal variation in telomere length of a hibernating rodent. Biol. Lett..

[49] Turbill C, Smith S, Deimel C, Ruf T (2012). Daily torpor is associated with telomere length change over winter in Djungarian hamsters. Biol. Lett..

[50] Armitage KB, Blumstein DT, Woods BC (2003). Energetics of hibernating yellow-bellied marmots (*Marmota flaviventris*). Comp. Biochem. Physiol. A.

[51] Armitage, K. B. in *Molecules to Migration: the Pressures of Life* (eds Morris, S. & Vosloo, A.) 591–602 (Medimond Publishing, 2008).

[52] Haghani, A. et al. DNA methylation networks underlying mammalian traits. Preprint at *bioRxiv*10.1101/2021.03.16.435708 (2021).

[53] Lu, A. T. et al. Universal DNA methylation age across mammalian tissues. Preprint at *bioRxiv*10.1101/2021.01.18.426733 (2021).

[54] Yang, S. et al. Rare mutations in *AHDC1* in patients with obstructive sleep apnea. *Biomed. Res. Int.*10.1155/2019/5907361 (2019). 10.1155/2019/5907361 PMC6815587 31737670

[55] De Paoli-Iseppi R (2017). Measuring animal age with DNA methylation: from humans to wild animals. Front. Genet..

[56] Arneson A (2022). A mammalian methylation array for profiling methylation levels at conserved sequences. Nat. Commun..

[57] Armitage KB (1998). Reproductive strategies of yellow-bellied marmots: energy conservation and differences between the sexes. J. Mammal..

[58] Armitage, K. B. in *Adaptive Strategies and Diversity in Marmots* (eds Ramousse, R. et al.) 133–142 (International Marmot Network, 2003).

[59] Snir S, Farrell C, Pellegrini M (2019). Human epigenetic ageing is logarithmic with time across the entire lifespan. Epigenetics.

[60] Snir S, VonHoldt BM, Pellegrini M (2016). A statistical framework to identify deviation from time linearity in epigenetic aging. PLoS Comput. Biol..

[61] Farrell C, Snir S, Pellegrini M (2020). The epigenetic pacemaker: modeling epigenetic states under an evolutionary framework. Bioinformatics.

[62] Marioni RE (2019). Tracking the epigenetic clock across the human life course: a meta-analysis of longitudinal cohort data. J. Gerontol. A.

[63] El Khoury LY (2019). Systematic underestimation of the epigenetic clock and age acceleration in older subjects. Genome Biol..

[64] Kilgore DL, Armitage KB (1978). Energetics of yellow-bellied marmot populations. Ecology.

[65] Armitage KB (1991). Social and population dynamics of yellow-bellied marmots: results from long-term research. Annu. Rev. Ecol. Syst..

[66] Webb DR (1980). Environmental harshness, heat stress, and *Marmota flaviventris*. Oecologia.

[67] Armitage KB (1999). Evolution of sociality in marmots. J. Mammal..

[68] Allainé D (2000). Sociality, mating system and reproductive skew in marmots: evidence and hypotheses. Behav. Processes.

[69] Arnold W (1990). The evolution of marmot sociality. II. Costs and benefits of joint hibernation. Behav. Ecol. Sociobiol..

[70] Villanueva-Cañas JL, Faherty SL, Yoder AD, Albà MM (2014). Comparative genomics of mammalian hibernators using gene networks. Integr. Comp. Biol..

[71] Lyman CP, O’Brien RC, Greene GC, Papafrangos ED (1981). Hibernation and longevity in the Turkish hamster *Mesocricetus brandti*. Science.

[72] Kirby R, Johnson HE, Alldredge MW, Pauli JN (2019). The cascading effects of human food on hibernation and cellular aging in free-ranging black bears. Sci. Rep..

[73] Giroud S (2014). Late-born intermittently fasted juvenile garden dormice use torpor to grow and fatten prior to hibernation: consequences for ageing processes. Proc. R. Soc. Lond. B.

[74] Hoelzl F (2016). Telomeres are elongated in older individuals in a hibernating rodent, the edible dormouse (*Glis glis*). Sci. Rep..

[75] Haussmann MF, Mauck RA (2008). Telomeres and longevity: testing an evolutionary hypothesis. Mol. Biol. Evol..

[76] van Lieshout SHJ (2019). Individual variation in early-life telomere length and survival in a wild mammal. Mol. Ecol..

[77] Lowe D, Horvath S, Raj K (2016). Epigenetic clock analyses of cellular senescence and ageing. Oncotarget.

[78] Kabacik S, Horvath S, Cohen H, Raj K (2018). Epigenetic ageing is distinct from senescence-mediated ageing and is not prevented by telomerase expression. Aging.

[79] Keil G, Cummings E, Magalhães JP (2015). Being cool: how body temperature influences ageing and longevity. Biogerontology.

[80] Means LW, Higgins JL, Fernandez TJ (1993). Mid-life onset of dietary restriction extends life and prolongs cognitive functioning. Physiol. Behav..

[81] Speakman JR, Mitchell SE (2011). Caloric restriction. Mol. Aspects Med..

[82] Walford RL, Spindler SR (1997). The response to calorie restriction in mammals shows features also common to hibernation: a cross-adaptation hypothesis. J. Gerontol. A.

[83] Conti B (2006). Transgenic mice with a reduced core body temperature have an increased life span. Science.

[84] Conti B (2008). Considerations on temperature, longevity and aging. Cell. Mol. Life Sci..

[85] Gribble KE, Moran BM, Jones S, Corey EL, Mark Welch DB (2018). Congeneric variability in lifespan extension and onset of senescence suggest active regulation of aging in response to low temperature. Exp. Gerontol..

[86] Johns DW, Armitage KB (1979). Behavioral ecology of alpine yellow-bellied marmots. Behav. Ecol. Sociobiol..

[87] Armitage KB (1962). Social behaviour of a colony of the yellow-bellied marmot (*Marmota flaviventris*). Anim. Behav..

[88] Armitage KB (1965). Vernal behaviour of the yellow-bellied marmot (*Marmota flaviventris*). Anim. Behav..

[89] Armitage KB, Melcher JC, Ward JM (1990). Oxygen consumption and body temperature in yellow-bellied marmot populations from montane-mesic and lowland-xeric environments. J. Comp. Physiol. B.

[90] Sheriff MJ, Williams CT, Kenagy GJ, Buck CL, Barnes BM (2012). Thermoregulatory changes anticipate hibernation onset by 45 days: data from free-living arctic ground squirrels. J. Comp. Physiol. B.

[91] Schwartz C, Hampton M, Andrews MT (2015). Hypothalamic gene expression underlying pre-hibernation satiety. Genes Brain Behav..

[92] Geiser F (2004). Metabolic rate and body temperature reduction during hibernation and daily torpor. Annu. Rev. Physiol..

[93] Maegawa S (2010). Widespread and tissue specific age-related DNA methylation changes in mice. Genome Res..

[94] Hampton M, Melvin RG, Andrews MT (2013). Transcriptomic analysis of brown adipose tissue across the physiological extremes of natural hibernation. PLoS ONE.

[95] Lindner M (2021). Temporal changes in DNA methylation and RNA expression in a small song bird: within- and between-tissue comparisons. BMC Genomics.

[96] Schwartz C, Hampton M, Andrews MT (2013). Seasonal and regional differences in gene expression in the brain of a hibernating mammal. PLoS ONE.

[97] Dopico XC (2015). Widespread seasonal gene expression reveals annual differences in human immunity and physiology. Nat. Commun..

[98] Jansen HT (2019). Hibernation induces widespread transcriptional remodeling in metabolic tissues of the grizzly bear. Commun. Biol..

[99] Viitaniemi HM (2019). Seasonal variation in genome-wide DNA methylation patterns and the onset of seasonal timing of reproduction in great tits. Genome Biol. Evol..

[100] Johnston RA, Paxton KL, Moore FR, Wayne RK, Smith TB (2016). Seasonal gene expression in a migratory songbird. Mol. Ecol..

[101] Boyer BB, Barnes BM (1999). Molecular and metabolic aspects of mammalian hibernation. Bioscience.

[102] Siutz C, Ammann V, Millesi E (2018). Shallow torpor expression in free-ranging common hamsters with and without food supplements. Front. Ecol. Evol..

[103] Langer F, Havenstein N, Fietz J (2018). Flexibility is the key: metabolic and thermoregulatory behaviour in a small endotherm. J. Comp. Physiol. B.

[104] Bieber C, Turbill C, Ruf T (2018). Effects of aging on timing of hibernation and reproduction. Sci. Rep..

[105] Storey KB, Storey JM (2012). Aestivation: signaling and hypometabolism. J. Exp. Biol..

[106] Krivoruchko A, Storey KB (2010). Forever young: mechanisms of natural anoxia tolerance and potential links to longevity. Oxid. Med. Cell. Longev..

[107] Storey KB, Storey JM (2004). Metabolic rate depression in animals: transcriptional and translational controls. Biol. Rev..

[108] Puspitasari A (2021). Hibernation as a tool for radiation protection in space exploration. Life.

[109] Blumstein DT (2013). Yellow-bellied marmots: insights from an emergent view of sociality. Philos. Trans. R. Soc. Lond. B.

[110] Armitage KB, Downhower JF (1974). Demography of yellow-bellied marmot populations. Ecology.

[111] Zhou W, Triche TJ, Laird PW, Shen H (2018). SeSAMe: reducing artifactual detection of DNA methylation by Infinium BeadChips in genomic deletions. Nucleic Acids Res..

[112] Labarre BA (2019). MethylToSNP: identifying SNPs in Illumina DNA methylation array data. Epigenetics Chromatin.

[113] Snir S, Wolf YI, Koonin EV (2012). Universal pacemaker of genome evolution. PLoS Comput. Biol..

[114] Zou H, Hastie T (2005). Regularization and variable selection via the elastic net. J. R. Stat. Soc. B.

[115] Friedman J, Hastie T, Tibshirani R (2010). Regularization paths for generalized linear models via coordinate descent. J. Stat. Softw..

[116] Snir S, Pellegrini M (2018). An epigenetic pacemaker is detected via a fast conditional expectation maximization algorithm. Epigenomics.

[117] Wood, S. & Scheipl, F. gamm4: Generalized additive mixed models using mgcv and lme4, R package version 0.2-3 (2014); http://cran.r-project.org/package=gamm4

[118] R Core Team. *R: A Language and Environment for Statistical Computing* (R Foundation for Statistical Computing, 2020).

[119] RStudio Team. *RStudio: Integrated Development Environment for R* (RStudio Inc., 2019).

[120] Van Rossum, G. & Drake, F. L. *Python 3 Reference Manual* (CreateSpace, 2009).

[121] Kluyver, T. et al. in *Positioning and Power in Academic Publishing: Players, Agents and Agendas* (eds Loizides, F. & Scmidt, B.) 87–90 (IOS Press, 2016); 10.3233/978-1-61499-649-1-87

[122] Wickham, H. *ggplot2: Elegant Graphics for Data Analysis* (Springer-Verlag, 2016).

[123] Kassambara, A. ggpubr: ‘ggplot2’ based publication ready plots https://cran.r-project.org/package=ggpubr (2020).

[124] Wood SN (2011). Fast stable restricted maximum likelihood and marginal likelihood estimation of semiparametric generalized linear models. J. R. Stat. Soc. B.

[125] Kuznetsova A, Brockhoff PB, Christensen RHB (2017). lmerTest package: tests in linear mixed effects models. J. Stat. Softw..

[126] Mclean CY (2010). GREAT improves functional interpretation of cis-regulatory regions. Nat. Biotechnol..

[127] Pinho, G. M. et al. Hibernation slows epigenetic ageing in yellow-bellied marmots data sets. *OSF*10.17605/OSF.IO/E42ZV (2021). 10.1038/s41559-022-01679-1 PMC8986532 35256811

