# Supplementary material for: Hibernation slows epigenetic ageing in yellow-bellied marmots
Source: Nat Ecol Evol. 2022 Mar 7;6(4):418–26. doi: 10.1038/s41559-022-01679-1 (PMC8986532; doi:10.1038/s41559-022-01679-1)
Supplement: Supplementary file 1 — Additional analysis evaluating the effect of the CpGs inputted in the EPM on GAMM results and testing the hibernation–ageing hypothesis with samples collected from same individuals across consecutive years. [file 41559_2022_1679_MOESM1_ESM.pdf]

nature portfolio

https://doi.org/10.1038/s41559-022-01679-1

## Supplementary information

# Hibernation slows epigenetic ageing in yellow-bellied marmots

In the format provided by the authors and unedited

Index

The effect of CpGs inputted in the EPM on GAMM results………. Page 1
Test of the hibernation-ageing hypothesis with samples collected from same individuals across consecutive years………. Page 2

## The effect of CpGs inputted in the EPM on GAMM results

The CpG sites used as input in the Epigenetic Pacemaker (EPM) model were selected based on their relationship with chronological age (we used only sites with an absolute Pearson correlation $r > 0.7$). Since this is a relatively arbitrary number, we tested whether a different criteria for the selection of sites would change our results. We estimated epigenetic states from EPMs including sites with a minimum absolute $r$ of 0.1, 0.5, 0.6, 0.7 or 0.8. The epigenetic states from each group were used as dependent variables in the GAMMs, such as performed in the manuscript. As shown in the table below, the effect of chronological age (cubic smoother spline) was significant in all models. The GAMMs with epigenetic states calculated from fewer sites (with high correlation with chronological age) are better at detecting the seasonal effect. By contrast, the influence of seasonality is lost in the GAMMs with epigenetic states calculated from many sites (including those sites that have lower correlations with chronological age).

| Minimum absolute *r* | | | 0.1 | 0.5 | 0.6 | 0.7 | 0.8 |
|---|---|---|---|---|---|---|---|
| | Intercept | Estimate | 5.43 | 5.51 | 5.51 | 5.53 | 5.61 |
| | | Std. Error | 0.27 | 0.13 | 0.10 | 0.09 | 0.10 |
| | | t value | 20.07 | 43.59 | 54.69 | 63.22 | 56.81 |
| | | p-value | <0.0001 | <0.0001 | <0.0001 | <0.0001 | <0.0001 |
| Smooth terms: | Age (cubic spline) | edf | 4.01 | 6.14 | 7.02 | 7.15 | 6.52 |
| | | Ref.df | 4.01 | 6.14 | 7.02 | 7.15 | 6.52 |
| | | F | 38.46 | 174.14 | 264.96 | 339.20 | 303.03 |
| | | p-value | <0.0001 | <0.0001 | <0.0001 | <0.0001 | <0.0001 |
| | Date (cyclic spline) | edf | 0.00 | 0.65 | 1.03 | 1.39 | 1.54 |
| | | Ref.df | 8.00 | 8.00 | 8.00 | 8.00 | 8.00 |
| | | F | 0.00 | 0.15 | 0.83 | 3.27 | 6.74 |
| | | p-value | 1.000 | 0.139 | **0.034** | **0.002** | **0.000** |
| Adjusted R-sq. | | | 0.55 | 0.90 | 0.94 | 0.96 | 0.94 |
| Residual variance | | | 5.54 | 1.15 | 0.46 | 0.29 | 0.23 |
| Number of CpG sites | | | 20,608 | 2,490 | 944 | 309 | 61 |

* The model with gray background was used in the main manuscript.

Test of the hibernation-ageing hypothesis with samples collected from same individuals across consecutive years

To provide additional evidence that epigenetic aging is slower during hibernation, we subsampled our data for the individuals with samples collected in consecutive years. We further restricted this subset for the cases where two or more samples were collected in one year and at least one other sample in the other year from the same individual. The samples chosen are highlighted in the figure below:

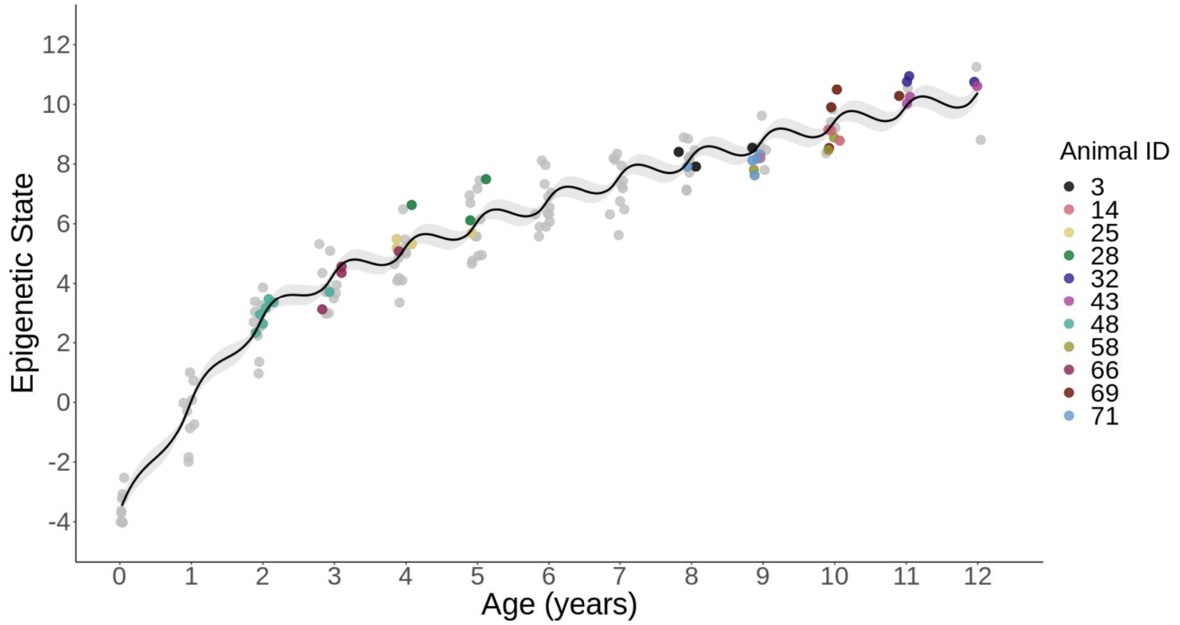


This figure is the same Figure 2A provided in the main manuscript file, with the samples used for this additional analysis in colour. For a better visualization, the plots below focus on the samples from young (< 7 years) and older individuals:

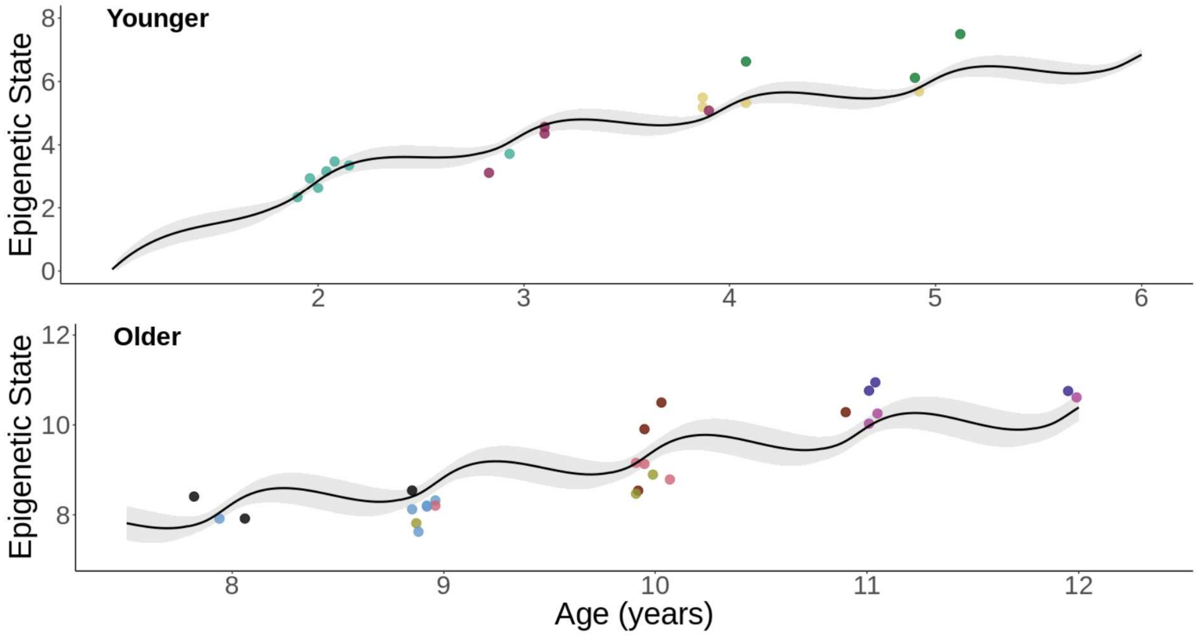

From this subset, we were able to estimate the rate of epigenetic aging per individual during the active and hibernation seasons. We only measured epigenetic ageing from epigenetic ages calculated from the Epigenetic pacemaker (EPM) model (described in the main manuscript file). To measure the active epigenetic ageing rate, we subtracted the epigenetic age from the latest sample by the earliest in the active season, and divided by the number of days in between the sampling dates. To measure the hibernation epigenetic ageing rate, we subtracted the epigenetic age of the last sample before hibernation from the epigenetic age from the first sample collected after hibernation, divided by the number of days between the collection events. The box plot with the epigenetic ageing rate per day is shown below:

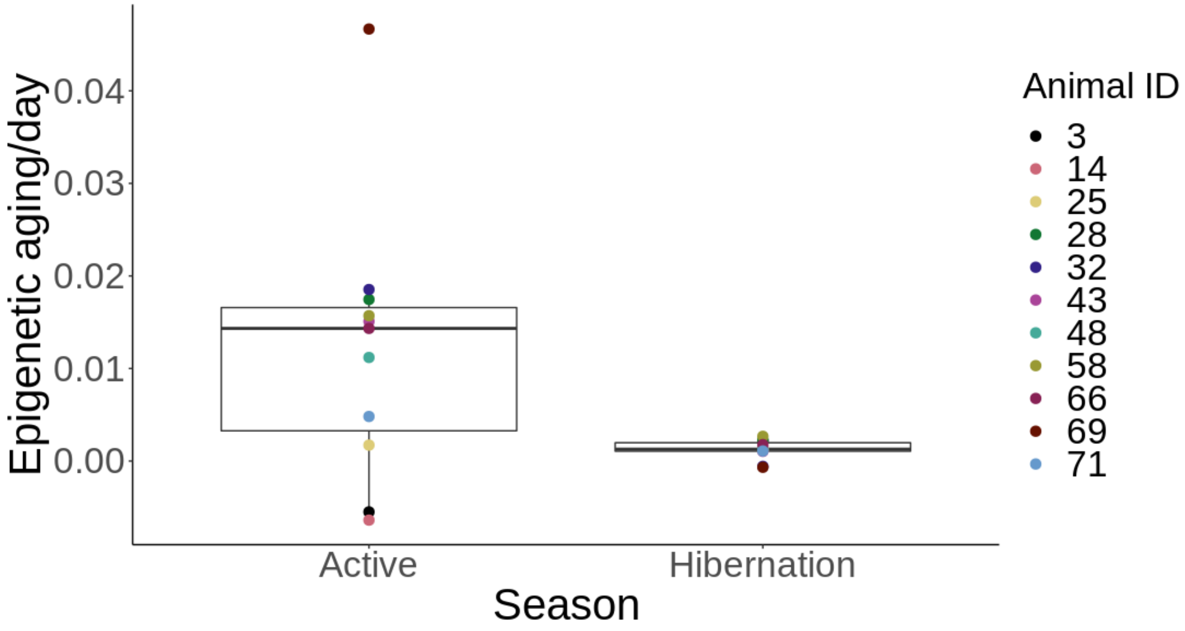


This is the same as Figure 3 in the main manuscript file, but with added information about individuals in colour. Interestingly, the individuals with negative epigenetic ageing rates (individuals 3 and 14) and the individual with the highest rate during the active season (ind. 69) are all old, which approximate ages of 8, 10 and 10, respectively. Our small sample size does not allow strong conclusions, however it would be interesting to evaluate whether older individuals show a higher variation in epigenetic ageing rates within active seasons.

With the same samples, we also analysed the average change in methylation levels across CpG sites during the active and hibernation seasons. First, we used the 309 CpGs inputted in the EPM model. The average change in methylation levels across these sites was higher during the active season than during hibernation (Kruskal-Wallis chi-squared = 15.783, df = 1, p-value = $7.105e^{-05}$), as seen below:

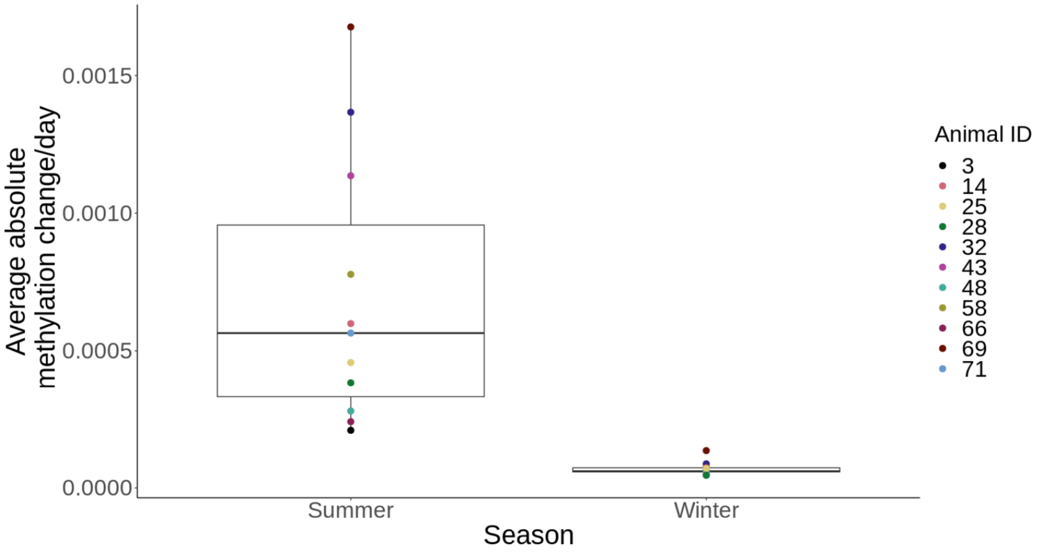


The average change in methylation levels across all sites from the mammalian array that mapped uniquely to CpG sites (and their respective flanking regions) in the yellow-bellied marmot assembly (31,388 CpGs) show a very similar pattern (Kruskal-Wallis chi-squared = 15.783, df = 1, p-value = $7.105e^{-05}$).

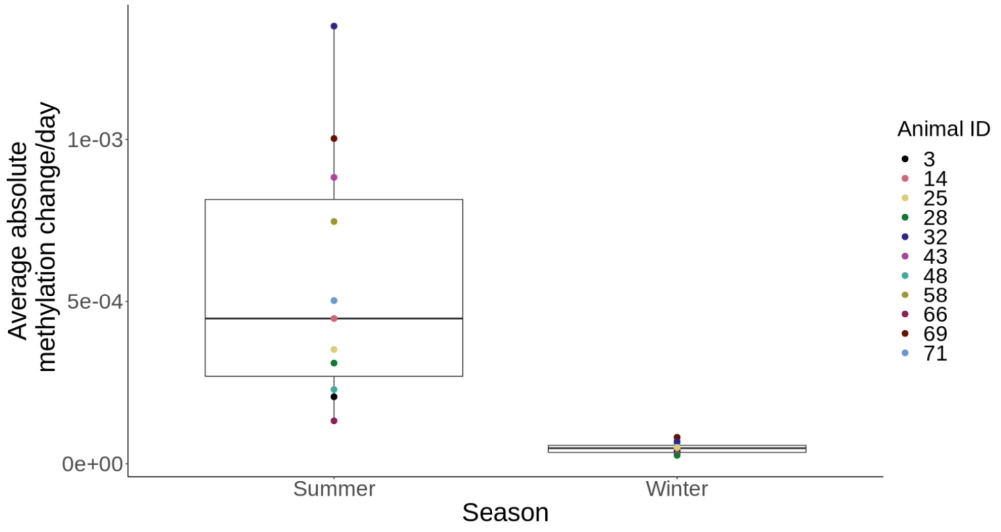


All the results from these additional analyses, focusing on individuals with samples from consecutive years, reinforce the main result in the manuscript that epigenetic ageing is significantly slower during

hibernation than during the active season. However, these additional plots and analysis have an important limitation. We compared changes in methylation in between individual captures, but these methylation changes are not necessarily ordered. It is possible that the methylation values for many of the sites are not accumulating in an ordered fashion. In this regard, the EPM model estimated epigenetic states from ordered methylation changes (with chronological age), and allowed us to use a much larger number of samples. The EPM results are therefore the main contribution for testing the hibernation-ageing hypothesis.

It is important to notice that the hibernation-ageing hypothesis does not require that age-unrelated CpGs are unaffected by hibernation. Hibernation substantially modifies several aspects of individual biology and metabolism, therefore changes at the epigenome are expected. The influence of hibernation on epigenetic ageing is evident because CpGs with high chronological-age predictive power show slower change rates during hibernation, and the influence of seasons on other biological processes may be equally possible. Horvath (2013) proposed that the epigenetic clock is a result of the cumulative effects of an epigenetic maintenance system (EMS). Such machinery would impact the epigenome as a whole and, assuming the EMS hypothesis holds, our results suggest that this machinery is suppressed in hibernating individuals.

The similar results among the 309 and 31k sites are not entirely surprising because the CpGs from the Mammalian array are not completely independent. Haghani et al. (2021) identified 55 co-methylation modules in the mammalian array using an unbiased clustering of individual citosines from 176 species. From those, 31 modules could be associated with life history traits. In our dataset, the average change in methylation levels computed for 309 and 31k CpGs were different, however, the correlation between them was 0.91. Given that we have only 22 observations for this analysis and the residuals are not normal when using a parametric test, we used a non-parametric test (Kruskall wallis sum-rank test). A test on ranks (instead of true values) of highly correlated data sets is expected to have similar results.

To further explore the relationships of the CpGs with chronological age and seasons, we subtracted the winter methylation change rate from the summer rate for each site per individual, and measured the mean difference in rates per site. These mean values were plotted against the CpGs' Pearson correlation values with chronological age, as shown below:

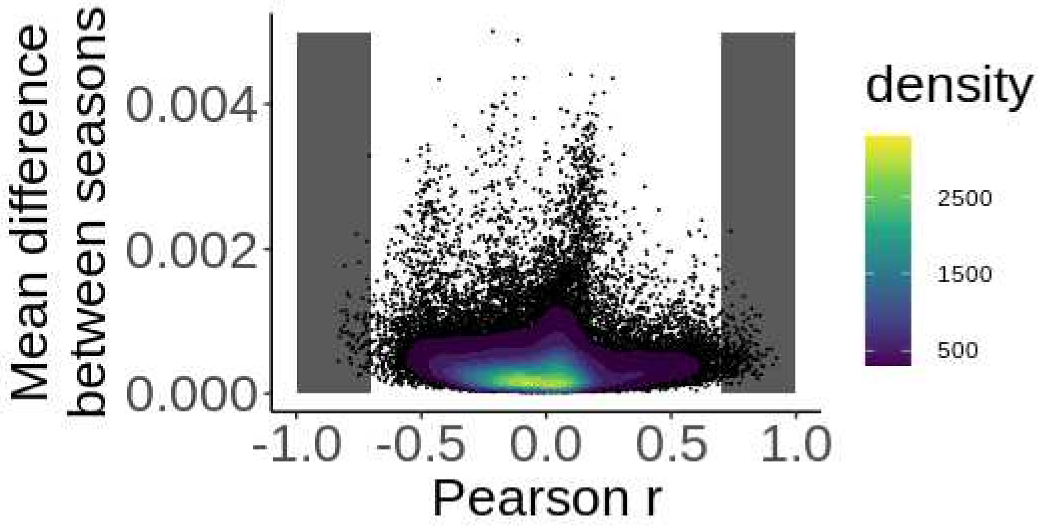


These plots cannot lead to strong conclusions because the measures per site were calculated from only 11 individuals, but a few patterns can be observed. The first plot shows that chronological age is associated with more CpGs than the 309 selected for the EPM, which are highlighted by the grey rectangles. Our cutoff for selecting sites to input in the EPM model was high (an minimum absolute *r* of 0.7), and, for example, were we to choose a threshold of 0.5, we would have developed the EPM with 2,490 sites. We show above (Page 1) that, even when CpGs that were weakly associated with age (absolute r > 0.1) were inputted in the EPM, the effect of chronological age was still detected in the GAMMs. Also, the CpGs inputted in the EPM are not the sites where the mean difference in methylation rates between seasons is the highest. There are many other sites, including sites with high and low Pearson *r* values with chronological age, that seem to be affected by seasonality. This helps explain why the results from the 309 sites are similar to the results from the 31k sites, and implies an epigenome-level effect of seasons.
