# Supplementary material for: Hibernation slows epigenetic ageing in yellow-bellied marmots
Source: Nat Ecol Evol. 2022 Mar 7;6(4):418–26. doi: 10.1038/s41559-022-01679-1 (PMC8986532; doi:10.1038/s41559-022-01679-1)
Supplement: Supplementary file 2 — Reporting Summary. [file 41559_2022_1679_MOESM2_ESM.pdf]

nature research

Corresponding author(s): Steve Horvath, Gabriela M. Pinho, Julien G. A. Martin, Daniel T. Blumstein

Last updated by author(s): Dec 31, 2021

Reporting Summary

Nature Research wishes to improve the reproducibility of the work that we publish. This form provides structure for consistency and transparency in reporting. For further information on Nature Research policies, see our Editorial Policies and the Editorial Policy Checklist.

Statistics

For all statistical analyses, confirm that the following items are present in the figure legend, table legend, main text, or Methods section.

n/a	Confirmed	
☐	☒	The exact sample size (*n*) for each experimental group/condition, given as a discrete number and unit of measurement
☐	☒	A statement on whether measurements were taken from distinct samples or whether the same sample was measured repeatedly
☐	☒	The statistical test(s) used AND whether they are one- or two-sided<br>*Only common tests should be described solely by name; describe more complex techniques in the Methods section.*
☐	☒	A description of all covariates tested
☐	☒	A description of any assumptions or corrections, such as tests of normality and adjustment for multiple comparisons
☐	☒	A full description of the statistical parameters including central tendency (e.g. means) or other basic estimates (e.g. regression coefficient) AND variation (e.g. standard deviation) or associated estimates of uncertainty (e.g. confidence intervals)
☐	☒	For null hypothesis testing, the test statistic (e.g. *F*, *t*, *r*) with confidence intervals, effect sizes, degrees of freedom and *P* value noted<br>*Give P values as exact values whenever suitable.*
☒	☐	For Bayesian analysis, information on the choice of priors and Markov chain Monte Carlo settings
☒	☐	For hierarchical and complex designs, identification of the appropriate level for tests and full reporting of outcomes
☐	☒	Estimates of effect sizes (e.g. Cohen's *d*, Pearson's *r*), indicating how they were calculated

Our web collection on statistics for biologists contains articles on many of the points above.

Software and code

Policy information about availability of computer code

Data collection	R_3.6.3: programming language for statistical computing<br>R_sesame_1.7.5: normalize Illumina Infinium DNA methylation array data<br>MethylToSNP_v.0.99.0: identify CpG sites that corresponded to SNPs<br>R_glmnet_v.4.0-2: fit a generalized linear model with elastic-net penalization<br>python_v.3.7.4: programming language<br>python_EpigeneticPacemaker_0.0.3: fit Epigenetic pacemaker model
Data analysis	R_v.3.6.3: programming language for statistical computing<br>R_gamm4_v.0.2-6: fit Generalized Additive Mixed Models<br>R_MuMIn_v.1.43.17: Model selection<br>R_tidymv_v3.2.1: used in simulations<br>R_tidyverse_v.1.3.1: used in simulations<br>R_patchwork_v.1.1.1: used in simulations<br>R_gridExtra_v.2.3: used in simulations<br>R_mgcv_v.1.8: Generalized Additive Models<br>R_lmerTest_v3.1-3: epigenome-wide association studies of age<br>R_ggplot2_v.3.3.0.9: visualization of Epigenetic aging models and the generalized additive mixed model<br>R_Genomic Regions Enrichment Tool_v.3.0.0: Gene enrichment

For manuscripts utilizing custom algorithms or software that are central to the research but not yet described in published literature, software must be made available to editors and reviewers. We strongly encourage code deposition in a community repository (e.g. GitHub). See the Nature Research guidelines for submitting code & software for further information.

Data

Policy information about availability of data

All manuscripts must include a data availability statement. This statement should provide the following information, where applicable:

- Accession codes, unique identifiers, or web links for publicly available datasets
- A list of figures that have associated raw data
- A description of any restrictions on data availability

Epigenetic data is deposited in the Gene Expression Omnibus GSE174544 and at http:://doi.org/10.17605/OSF.IO/E42ZV
Code and all supplementary files are available at http:://doi.org/10.17605/OSF.IO/E42ZV

Field-specific reporting

Please select the one below that is the best fit for your research. If you are not sure, read the appropriate sections before making your selection.

☐ Life sciences ☐ Behavioural & social sciences ☒ Ecological, evolutionary & environmental sciences

For a reference copy of the document with all sections, see nature.com/documents/nr-reporting-summary-flat.pdf

Ecological, evolutionary & environmental sciences study design

All studies must disclose on these points even when the disclosure is negative.

Study description	We tested the hypothesis that aging is suspended during hibernation in a well-studied population of yellow-bellied marmots (Marmota flaviventer), which spend 7-8 months per year hibernating. We used two approaches to estimate epigenetic age: the epigenetic clock and the epigenetic pacemaker. Variation in epigenetic age of 149 samples collected throughout the life of 73 females was modeled using generalized additive mixed models (GAMM), where season (cyclic cubic spline) and chronological age (cubic spline) were fixed effects.
Research sample	All samples were collected as part of a long-term study of a free-living population of yellow-bellied marmots in the Gunnison National Forest, Colorado (USA), where marmots were captured and blood samples collected biweekly during their active season (May to August). Individuals were monitored throughout their lives, and chronological age was calculated based on the date at which juveniles first emerged from their natal burrows. We only used female samples because precise age for most adult males is unavailable since males are typically immigrants born elsewhere.
Sampling strategy	We selected 160 blood samples from 78 females with varying ages. From these, DNA methylation profiling passed quality control for 149 samples from 73 females with ages varying from 0.01 to 12.04 years. We used two simulations approaches to estimate the type 1 error and the power to detect a hibernation-ageing effect given the limitations of our sample collection. Specifically, blood samples could only be collected during the active season, instead of throughout the year. Our earliest sample was collected on 27 April and the latest on 20 August. In our first approach, we simulated two traits (Figure S1): (1) a trait that increases linearly with age independently of the season; and (2) a trait that increases during the summer but not during the winter. The daily rate of increase for the first trait was set at 0.004, to simulate data with a similar range to the observed EPM data. For the second trait, the rate of increase was set to zero during winter (16 Sept to 17 April, days 139-352 using 1 May as reference). The simulation assumed that the active season was 150 days long starting on 18 April (day 353) and finishing on 15 Sept (day 138). The rate of increase during the active season was set as 0.0164 (0.004 / 365 * 150) so that the annual rate of increase was similar between the two simulated traits. Our simulation was parametrized using among-individual and residual variance from the EPM. We performed these simulations using field data (day of sample collection, age in days, birth date, and number of samples), and estimated the significance of the seasonal effect with the GAMM that best explained the marmot data (model selection described above). We repeated this procedure 1,000 times for both traits. The proportion of simulations on trait 1 (no seasonal effect) that were significant indicated our type 1 error. The proportion of simulations on trait 2 (seasonal effect) that were significant was an indication of the power to detect this effect. In the second approach, we simulated 1,000 data sets where day of year was randomly shuffled. The type 1 error was estimated as the proportion of simulations with significant seasonal effects in the GAMMs.
Data collection	Since the data came from a long-term study, many generations of postdoctoral researchers, grad students, research assistants and undergrads have collected samples. Since 2002, Professor Daniel Blumstein has been the leader of the project, and all members of the field team are trained on animal trapping, sample collection and animal observation prior to starting field activities.
Timing and spatial scale	The samples used in this manuscript have been collected from 2004 to 2018. The free living yellow-bellied marmots in the Gunnison National Forest, Colorado (USA), are studied every year from May to August.
Data exclusions	We selected 160 blood samples from 78 females with varying ages. We used two different unsupervised hierarchical clustering procedures to identify technical outliers. The first clustering procedure was based on imputed SNPs. Toward this end, we used MethylToSNP v.0.99.0 to identify CpG sites that corresponded to SNPs. The SNP data was used for unsupervised hierarchical clustering based on Euclidean distances. Branches (clusters) of the cluster tree corresponded to multiple samples from the same animal. This allowed us to identify a small platemap error probably caused by human pipetting error. To err on the side of caution we removed putative platemap errors from the data set. Second, we carried out average linkage hierarchical clustering based on the inter-array correlation to identify technical outliers due to an insufficient amount of DNA. The DNAm profiling from 149 samples passed quality control. These samples were collected from 73 females (1 to 8 samples per individual) with ages varying from 0.01 to 12.04 years.

Reproducibility	All the DNA methylation data, detailed sample information, and code have been made available.
Randomization	The order in which blood samples have been extracted for DNA and analyzed with the methylation array have been randomized.
Blinding	DNA extraction and methylation array have been performed by individuals without knowledge about the samples.

Did the study involve field work? ☒ Yes ☐ No

Field work, collection and transport

Field conditions	The study was performed in a highly seasonal environment, where marmots hibernate from 7 to 8 months per year. Individuals reside in either 'up-valley' or 'down-valley' colonies that differ by an elevational gradient of 165 m.
Location	The wild population of yellow-bellied marmots is located in and around the Rocky Mountain Biological Laboratory (38°57'N, 106° 59'W; 2900 m elevation) in Colorado, USA.
Access & import/export	The field site is located in a National Forest, and it is accessible by car, bike or foot. Data and samples were collected under the UCLA Institutional Animal Care and Use protocol (2001-191-01, renewed annually) and with permission from the Colorado Parks and Wildlife (TR917, renewed annually).
Disturbance	We prioritize animal wellbeing by minimizing the time individuals spend within traps, particularly in warm days. In areas close to trails used by park visitors, one member of the team monitors the traps to avoid any interaction between dogs and trapped animals. The traps are removed from animals' path when they are not being used.

Reporting for specific materials, systems and methods

We require information from authors about some types of materials, experimental systems and methods used in many studies. Here, indicate whether each material, system or method listed is relevant to your study. If you are not sure if a list item applies to your research, read the appropriate section before selecting a response.

Materials & experimental systems

n/a	Involved in the study
☒	☐ Antibodies
☒	☐ Eukaryotic cell lines
☒	☐ Palaeontology and archaeology
☐	☒ Animals and other organisms
☒	☐ Human research participants
☒	☐ Clinical data
☒	☐ Dual use research of concern

Methods

n/a	Involved in the study
☒	☐ ChIP-seq
☒	☐ Flow cytometry
☒	☐ MRI-based neuroimaging

Animals and other organisms

Policy information about studies involving animals; ARRIVE guidelines recommended for reporting animal research

Laboratory animals	The study did not involve laboratory animals.
Wild animals	All samples were collected as part of a long-term study of a free-living population of yellow-bellied marmots (Marmota flaviventer). Trapping sessions occur biweekly from May to August (details in Blumstein et al., 2016). We trapped marmots with Tomahawk live traps (Tomahawk Live Trap Co., Tomahawk, WI) baited with Purina Omolene 100 Horse Feed (Purina Mills, LLC, Gray Summit, MO). All marmots are given two uniquely numbered metal ear tags (Monel self-piercing fish tags #3, National Band and Tag, Newport, KY, United States) for permanent identification and their dorsal pelage is marked with black Nyanzol fur dye to enable identification from afar (Blumstein 2013). We recorded individual sex, time and date of capture, and collected blood samples based on protocol approved by UCLA Institutional Animal Care and Use protocol (2001-191-01, renewed annually) and the Colorado Parks and Wildlife (TR917, renewed annually). Our goal was to minimize animal handling time. All handling was performed at the site of capture and no anesthesia was used, so animals were released shortly after handling.<br><br>Weather permitting, we performed daily behavioral observations during times of peak activity (0700-1000, and 1600-1900 h; Armitage, 1962). Observers used binoculars and 15-45x spotting scopes (Blumstein et al., 2009; Yang et al., 2017) to record individual identity and location.
Field-collected samples	We used 149 blood samples from 73 females with ages varying from 0.01 to 12.04 years. Samples were collected at the capture site and animals were released shortly after sample collection.
Ethics oversight	UCLA Institutional Animal Care and Use protocol (2001-191-01, renewed annually) and Colorado Parks and Wildlife (TR917, renewed annually).

Note that full information on the approval of the study protocol must also be provided in the manuscript.
