# Supplementary material for: Hibernation slows epigenetic ageing in yellow-bellied marmots
Source: Nat Ecol Evol. 2022 Mar 7;6(4):418–26. doi: 10.1038/s41559-022-01679-1 (PMC8986532; doi:10.1038/s41559-022-01679-1)
Supplement: Supplementary file 3 — Peer Review Information. [file 41559_2022_1679_MOESM3_ESM.pdf]

nature research

Peer Review Information

Journal: Nature Ecology & Evolution
Manuscript Title: Hibernation slows epigenetic ageing in yellow-bellied marmots
Corresponding author name(s): Gabriela M. Pinho, Daniel T. Blumstein, Steve Horvath

Editorial Notes:

Reviewer Comments & Decisions:

Decision Letter, initial version:

7th July 2021

*Please ensure you delete the link to your author homepage in this e-mail if you wish to forward it to your co-authors.

Dear Dr Horvath,

Your manuscript entitled "Hibernation slows epigenetic aging in yellow-bellied marmots" has now been seen by three reviewers, whose comments are attached. The reviewers have raised a number of concerns which will need to be addressed before we can offer publication in Nature Ecology & Evolution. We will therefore need to see your responses to the criticisms raised and to some editorial concerns, along with a revised manuscript, before we can reach a final decision regarding publication.

I should stress that we will need to see agreement among the reviewers for a robust demonstration that hibernation slows ageing in marmots.

We therefore invite you to revise your manuscript taking into account all reviewer and editor comments. Please highlight all changes in the manuscript text file in Microsoft Word format.

We are committed to providing a fair and constructive peer-review process. Do not hesitate to contact us if there are specific requests from the reviewers that you believe are technically impossible or unlikely to yield a meaningful outcome.

When revising your manuscript:

* Include a “Response to reviewers” document detailing, point-by-point, how you addressed each reviewer comment. If no action was taken to address a point, you must provide a compelling argument. This response will be sent back to the reviewers along with the revised manuscript.

* If you have not done so already please begin to revise your manuscript so that it conforms to our Article format instructions at http://www.nature.com/natecolevol/info/final-submission. Refer also to any guidelines provided in this letter.

* Include a revised version of any required reporting checklist. It will be available to referees (and, potentially, statisticians) to aid in their evaluation if the manuscript goes back for peer review. A revised checklist is essential for re-review of the paper.

Please use the link below to submit your revised manuscript and related files:

[REDACTED]

Note: This URL links to your confidential home page and associated information about manuscripts you may have submitted, or that you are reviewing for us. If you wish to forward this email to co-authors, please delete the link to your homepage.

We hope to receive your revised manuscript within four to eight weeks. If you cannot send it within this time, please let us know. We will be happy to consider your revision so long as nothing similar has been accepted for publication at Nature Ecology & Evolution or published elsewhere.

Nature Ecology & Evolution is committed to improving transparency in authorship. As part of our efforts in this direction, we are now requesting that all authors identified as ‘corresponding author’ on published papers create and link their Open Researcher and Contributor Identifier (ORCID) with their account on the Manuscript Tracking System (MTS), prior to acceptance. ORCID helps the scientific community achieve unambiguous attribution of all scholarly contributions. You can create and link your ORCID from the home page of the MTS by clicking on ‘Modify my Springer Nature account’. For more information please visit please visit www.springernature.com/orcid.

Please do not hesitate to contact me if you have any questions or would like to discuss these revisions further.

We look forward to seeing the revised manuscript and thank you for the opportunity to review your work.

[REDACTED]

Reviewer expertise:

Reviewer #1: physiology of hibernation

Reviewer #2: genomics in natural populations, epigenetic ageing

Reviewer #3: DNA methylation and aging, computational

Reviewers' comments:

Reviewer #1 (Remarks to the Author):

In this MS, the authors use an epigenetic clock based on DNA methylation to assess the 'biological age' of hibernators. At first glance and as a hibernation researcher, I found the study to be interesting, informative, and exceptionally novel. Essentially, we've known that hibernators may be very long lived for animals of their body mass e.g. some species of bats that are known to hibernate may live into their 30s. In this MS, the authors provide some of the first data I've seen that explores putative mechanism.

I have some issues that need to be addressed. I think my real question is that are the authors autocorrelating? In Fig. S2, examples of CpG sites that do not relate to chronological age are provided. As I understand the approach, the authors selected for sites that did relate to chronological age e.g. with a large enough data set, isn't it possible to find a relationship that fits the model? I am hoping another reviewer can better address this issue than I can. I think the results are extraordinary if they are mechanistically sound. I'd just like some independent assurance to that effect.

Should the above issue be satisfactorily resolved, my other issues are minor:

Not all hibernators are long lived and I would think the authors may wish to look at some of the shorter lived species (typically non-boreoeutherians like Sminthopsis). Such a study is out of the scope of this project and I am not suggesting its inclusion here.

Claudia Bieber, Chris Turnbill, and Thomas Ruf published a paper in 2018 (Scientific Reports) that should be addressed. Therein, the authors demonstrated an interrelationship with hibernation, reproduction, and aging and all the more complicated by resource availability during mast years. The reason I say this is that marmots live a pretty charmed life at RMBL (study site for the marmots here). Temperatures are cold and marmots are big. This means that hibernation is fairly 'predictable' if you will. Meaning that if you were to take a hibernator that lived in a more variable climate, would there be a breakdown in the reliability of the EC and EPM? Presumably, 'aging' would vary more by year. If this were to occur, what might that mean?

The authors used only female marmots. This is appropriate. Male marmots oftentimes are displaced and move around significantly making the desired data analyses here pretty much impossible if males had been included.

Lines 355-363 I'd argue against much of what the authors contend here. While there is a profound leukocytopenia and depression of immune function during torpor, the authors are aware that hibernation consists of both torpor and the intervening interbout arousals e.g. immune function is depressed for a 'short period' in a molecular timing. What I mean by this is that Annika Herwig working out of Paul Pevet's lab demonstrated Bmal, TIM, and PER oscillations are such that a one week torpor bout is really a single circadian day at a molecular scale. Similarly, we know that carbohydrate metabolism dominates in the interbout arousal and since ~70% of seasonal energy

stores are consumed in these IBAs, the majority of energy use is still carbohydrate based and not lipid based as the authors sort of imply. I think a little revision here would go a long way towards clearing up issues for a wider audience.

Reviewer #2 (Remarks to the Author):

In this paper, Pinho et al. generate DNA methylation measures in a well-studied population of marmots using a conserved mammalian DNA methylation chip. They use the resulting data to investigate the relationship between hibernation and the aging process. The dataset is unique and likely to be of interest to researchers working in ecological/evolutionary epigenetics. Additionally, a better understanding of the relationship between aging and hibernation has implications for the evolution of both, and so the analysis is intriguing and important. However, I have concerns about the strength of the evidence for the main claim that hibernation slows/stalls aging, which may stem from a statistical artifact.

Major comments:

The authors report a putative halting, or slowing, of aging during hibernation. This result is illustrated in Figure 2b, where a cyclic cubic spline relating day of sample to epigenetic state appears to decrease during the hibernating months and increase during the months when awake. The authors cannot sample during the hibernating months, which is an understandable constraint, and so this inference must be made solely from samples collected during non-hibernating months. However the cyclic cubic splines, by construction, must meet at the end points of the spline (e.g. days 1 and 365). As a result, if you have samples where individuals are getting older throughout the waking year (and so epigenetic state will tend to increase), in times of year when you don't have samples (i.e., hibernation), the spline will fit a decreasing trend to meet those constraints. Thus, the result in figure 2b could be a consequence of the usage of cyclic cubic splines, combined with the absence of samples during hibernation, rather than evidence for a slowing of aging itself.

The authors perform simulations for power analyses and false positive rate estimation, which in principle should address this problem. However, in trying to recreate these simulations from the R code provided, I was unable to complete more than a small number of simulations (e.g. <30) before the simulations failed due to singular convergence issues. As a result, I was unable to interpret or replicate the power/false positive rate estimates in the main text.

Given these concerns, the authors should consider other ways to use their dataset to get at the hibernation-aging hypothesis given their sampling constraints. One alternative analysis would be to focus on the sets of samples closest to the start and end of hibernation. The authors' hypothesis predicts that partial epigenetic states directly after the end hibernation should be lowest (as their chronological age has continued to increase while their epigenetic state has been under hibernation-induced stasis). As a result, comparisons of partial epigenetic states just prior to entering hibernation should be significantly higher, on average, than those for animals just emerging (in the subsequent year). Additionally, repeated measures within the same individuals could reveal how an individual's epigenetic trajectory changes longitudinally across seasons (also, please clarify how many repeated samples were available for individuals in the data set). Finally, it would be helpful to see direct, quantitative comparisons that show that more complex nonlinear/spline fits actually significantly

improve over simple linear fits (e.g., through comparing generalization error in predicting out-of-sample, formal model comparison, etc.). These should be applied both to the overall epigenetic clock/EPM analyses and to the site-by-site analyses. If all three of these analyses continue to support the manuscript's claims, the results would be much more convincing.

Finally, the authors perform gene set enrichment for effects of day of year/seasonality. They report a number of significant gene sets, which is surprising given the small number of significantly associated CpG cites (n=126). I am concerned that these results stem from poorly calibrated tests where only a small number of sites are even associated with a given functional term. I suggest removing terms with small numbers of tested sites (e.g., less than 10 or 20) from this analysis. The MHT could also be minimized by pruning out gene sets of uninterpretable functon(e.g., "HP:0002714: Downturned corners of mouth"); currently, over 60000 different gene sets are tested, as they are unlikely to provide any real biological insight.

Minor comments:

Lines 85: What quality control was performed to exclude the 11 samples?

Lines 226: Why was this significance threshold chosen?

Lines 296-297: Non-linear relationships between age and DNA methylation might be a product of technical effects (e.g. noise in methylation estimates, small sample size) or a true non-linear, biological relationship. The fact that 66% of age-associated sites had an EDF>2 could be consistent with either, but is not necessarily definitive effective of non-linear aging (especially since DNA methylation levels are bounded at 0 and 1). I'd suggest that the authors pull back on this conclusion throughout the manuscript.

Reviewer #3 (Remarks to the Author):

Pinhoa et al., have developed an epigenetic clock on yellow-bellied marmots. The authors have also developed an epigenetic pacemaker to assist in explaining the increase longevity in animals that hibernate. The work presented provides an interesting case study for epigenetic clocks and animals that hibernate, which has not been previously explored. As the authors discuss in the manuscript, epigenetic clocks may not be able to detect or capture seasonal variations due to the penalised regression. This may have implications in other species that experience cyclic patterns other than hibernation. Although, this study is well detailed and presented, I do have some minor suggestions and comments that the authors may be able to address.
Comments
Line 240. The authors report polycomb complex targets were proximal to the CpG sites and this pattern is consistent with other mammals. The authors reference the manuscript on bioRxiv.org titled 'Universal DNA methylation age across mammalian tissues.' This study referenced and the one presented both use the Illumina chip HorvathMammalMethylChip40. This array is a useful tool to develop epigenetic clocks for mammals as it can be applied to broad range of species.
My concern is that due to the selection of CpG sites in the Illumina array, the sites used for an epigenetic clock under this system will always be enriched for polycomb complex targets and pathways relating to development and cell differentiation. If the authors used a non-targeted approach to selecting CpG sites such as reduced representation bisulphite sequencing, such as the method used

in mice, would the CpG sites still be enriched for polycomb complex targets?
Mouse DNA methylation age study: https://pubmed.ncbi.nlm.nih.gov/28399939/
Minor comments:
Line 123. Were sites with a negative correlation also included?
Line 127. As a suggestion, 1-2 sentences of why a Generalized Additive Mixed Model was used may help readers who are not familiar with the method.
Line 180. Figure 1 shows an impressive epigenetic clock. Can the authors also provide the median absolute error rate and relative error?
Line 239. 'Most CpGs in promoter regions were hypermethylated with age' – can the specific number be provided?
Even more minor:
1. Should the gene names in the manuscript be italicised?

********************END********************

Author Rebuttal to Initial comments

natureresearch

Reviewer #1 (Remarks to the Author):

In this MS, the authors use an epigenetic clock based on DNA methylation to assess the 'biological age' of hibernators. At first glance and as a hibernation researcher, I found the study to be interesting, informative, and exceptionally novel. Essentially, we've known that hibernators may be very long lived for animals of their body mass e.g. some species of bats that are known to hibernate may live into their 30s. In this MS, the authors provide some of the first data I've seen that explores putative mechanism.

I have some issues that need to be addressed. I think my real question is that are the authors autocorrelating? In Fig. S2, examples of CpG sites that do not relate to chronological age are provided. As I understand the approach, the authors selected for sites that did relate to chronological age e.g. with a large enough data set, isn't it possible to find a relationship that fits the model? I am hoping another reviewer can better address this issue than I can. I think the results are extraordinary if they are mechanistically sound. I'd just like some independent assurance to that effect.

Response: It is a valid concern that a high dimensional data set coupled with a non-linear modeling technique may give rise to spurious patterns. We address this concern in several ways.

First, we now present several additional analyses to further illustrate the strength of our main results. Specifically, as requested by reviewer 2, we used model selection to test whether the model including the cyclic smooth term for day of year better explains our data than simpler models. The methods and results were updated accordingly (results in lines 92-114 and methods in lines 342-353). We improved the code for the simulation of traits with and without seasonal variation, used to estimate our type 1 error and analysis power. Moreover, we performed a second calculation of the type 1 error by simulating data where day of year was randomly shuffled (methods in lines 371-373). All approaches reinforced that our results were not driven by our sampling nor by statistical artifact (lines 115-130); thus, our results are statistically sound.

Second, Figure S2 showed examples of CpG sites that have a non-linear relationship with chronological age. We provided this figure to permit readers to observe changes in methylation in individual CpG sites over time. Indeed, we selected sites that were highly correlated with chronological age to develop the epigenetic pacemaker (EPM) model. The epigenetic state calculated by the EPM is a value per sample that minimizes the differences between known and estimated methylation levels for all pre-selected CpG sites in a given sample. Because the epigenetic state is a unique value per sample and is calculated from all inputted sites, it summarizes the overall trend across the methylome. Thus, the number of sites used to calculate epigenetic states does not necessarily increase the chance of finding a specific relationship. As shown in the table below, the GAMMs with epigenetic states calculated from fewer sites (with high correlation with chronological age) are better at detecting the seasonal effect. By contrast, the influence of seasonality is lost in the GAMMs with epigenetic states calculated from many sites (including those sites that have lower correlations with chronological age). We added the following table to Appendix A in the Supplementary material.

Minimum absolute *r*		0.1	0.5	0.6	0.7	0.8
Intercept	Estimate	5.43	5.51	5.51	5.53	5.61
	Std. Error	0.27	0.13	0.10	0.09	0.10
	t value	20.07	43.59	54.69	63.22	56.81
	p-value	<0.0001	<0.0001	<0.0001	<0.0001	<0.0001
Smooth terms: Age (cubic spline)	edf	4.01	6.14	7.02	7.15	6.52
	Ref.df	4.01	6.14	7.02	7.15	6.52
	F	38.46	174.14	264.96	339.20	303.03
	p-value	<0.0001	<0.0001	<0.0001	<0.0001	<0.0001
Smooth terms: Date (cyclic spline)	edf	0.00	0.65	1.03	1.39	1.54
	Ref.df	8.00	8.00	8.00	8.00	8.00
	F	0.00	0.15	0.83	3.27	6.74
	p-value	1.000	0.139	**0.034**	**0.002**	**0.000**
Adjusted R-sq.		0.55	0.90	0.94	0.96	0.94
Residual variance		5.54	1.15	0.46	0.29	0.23
Number of CpG sites		20,608	2,490	944	309	61

* The model with gray background was used as base for simulations and analysis per site.

Should the above issue be satisfactorily resolved, my other issues are minor:

Not all hibernators are long lived and I would think the authors may wish to look at some of the shorter lived species (typically non-boreoeutherians like Sminthopsis). Such a study is out of the scope of this project and I am not suggesting its inclusion here.

Response: This is certainly a valid point and we have reworded our text to avoid implying that all hibernators have long life. Wording was changed in line 25 “Species that hibernate generally live longer than would be expected based solely on their body size”, and line 272 “long lifespan seen in most hibernators”.

Claudia Bieber, Chris Turnbill, and Thomas Ruf published a paper in 2018 (Scientific Reports) that should be addressed. Therein, the authors demonstrated an interrelationship with hibernation, reproduction, and aging and all the more complicated by resource availability during mast years. The reason I say this is that marmots live a pretty charmed life at RMBL (study site for the marmots here). Temperatures are cold and marmots are big. This means that hibernation is fairly ‘predictable’ if you will. Meaning that if you were to take a hibernator that lived in a more variable climate, would there be a breakdown in the reliability of the EC and EPM? Presumably, 'aging' would vary more by year. If this were to occur, what might that mean?

Response: Thank you for suggesting this interesting article. We agree that epigenetic aging patterns should be different in mammals that vary more in their hibernation phenotype. Please note that we conclude by noting how the exploration of intra- and inter-specific variation in torpor use by future studies would potentially increase our knowledge about the mechanisms behind slower epigenetic aging during hibernation. There is an incredible variety in life strategies to survive freezing, apoxic or extremely hot environments that involve torpor, and not only edible dormice. Indeed, other vertebrates and even invertebrate species can and should be studied to uncover the links between hibernation/torpor, aging and epigenetics. In this regard, we would prefer to keep our conclusion broad

and not delve in this dormice study or in the fitness trade-offs between reproduction and hibernation. We added a citation of Bieber et al. (2018) in line 274.

We agree that species located in less-predicable environments will likely exhibit a higher variation in epigenetic aging rate per year, and we believe that some epigenetic aging variation should also occur in yellow-bellied marmots. Marmots that experience a harsh active season should age faster than individuals in optimal conditions. To answer such questions we need a larger sample size, and we are working on it. Other researchers with access to samples from individuals throughout hibernation would be in a great position to estimate the effect of different aspects of hibernation on epigenetic aging, such as torpor bout length and torpor-arousal transition frequency. Although this topic is of ultimate interest to us, this detailed discussion is out of the scope of the manuscript, where we are showing the overall trend that epigenetic aging slows during hibernation. Our discussion already touches on many of the points stated here.

The authors used only female marmots. This is appropriate. Male marmots oftentimes are displaced and move around significantly making the desired data analyses here pretty much impossible if males had been included.

Response: This was indeed our justification for focusing on females.

Lines 355-363 I'd argue against much of what the authors contend here. While there is a profound leukocytopenia and depression of immune function during torpor, the authors are aware that hibernation consists of both torpor and the intervening interbout arousals e.g. immune function is depressed for a 'short period' in a molecular timing. What I mean by this is that Annika Herwig working out of Paul Pevet's lab demonstrated Bmal, TIM, and PER oscillations are such that a one week torpor bout is really a single circadian day at a molecular scale. Similarly, we know that carbohydrate metabolism dominates in the interbout arousal and since ~70% of seasonal energy stores are consumed in these IBAs, the majority of energy use is still carbohydrate based and not lipid based as the authors sort of imply. I think a little revision here would go a long way towards clearing up issues for a wider audience.

Response: Excellent points; we agree that we focused on torpor without mentioning the interbout arousals. However, we changed this analysis based on suggestions from reviewer 2 and these biological functions are no longer significant (see below). We removed the paragraph containing lines 260-268 (previous lines 355-363) from the manuscript.

Reviewer #2 (Remarks to the Author):

In this paper, Pinho et al. generate DNA methylation measures in a well-studied population of marmots using a conserved mammalian DNA methylation chip. They use the resulting data to investigate the relationship between hibernation and the aging process. The dataset is unique and likely to be of interest to researchers working in ecological/evolutionary epigenetics. Additionally, a better understanding of the relationship between aging and hibernation has implications for the evolution of both, and so the analysis is intriguing and important. However, I have concerns about the strength of the evidence for the main claim that hibernation slows/stalls aging, which may stem from a statistical artifact.

Major comments:

The authors report a putative halting, or slowing, of aging during hibernation. This result is illustrated in Figure 2b, where a cyclic cubic spline relating day of sample to epigenetic state appears to decrease during the hibernating months and increase during the months when awake. The authors cannot sample during the hibernating months, which is an understandable constraint, and so this inference must be made solely from samples collected during non-hibernating months. However the cyclic cubic splines, by construction, must meet at the end points of the spline (e.g. days 1 and 365). As a result, if you have samples where individuals are getting older throughout the waking year (and so epigenetic state will tend to increase), in times of year when you don't have samples (i.e., hibernation), the spline will fit a decreasing trend to meet those constraints. Thus, the result in figure 2b could be a consequence of the usage of cyclic cubic splines, combined with the absence of samples during hibernation, rather than evidence for a slowing of aging itself.

The authors perform simulations for power analyses and false positive rate estimation, which in principle should address this problem. However, in trying to recreate these simulations from the R code provided, I was unable to complete more than a small number of simulations (e.g. <30) before the simulations failed due to singular convergence issues. As a result, I was unable to interpret or replicate the power/false positive rate estimates in the main text.

Response: Thank you for raising these issues; we believe that by addressing them the paper is now stronger. It is correct that the cyclic cubic spline function meet at the beginning and end of each cycle (in this case, a year), but it does not force a relationship that does not exist. In other words, if the change in epigenetic aging was linear throughout the year, the age cubic spline would capture this linear increase and the cyclic seasonal spline would simply be flat. See Figure S1 for an illustration extracted from our simulations. We apologize for any problem encountered during replication of our simulations since everything worked well on our computers. However, we should acknowledge that the simulations were often providing a warning due to the lack of variation associated to the random effect. The function "gamm" used in the simulation is quite sensitive to small variance estimate in random effects and tend to produce frequent warnings. We updated the code using a new package (gamm4 based on lme4 instead of mgcv). This package and function is much more robust to small variance estimates and did not produced any warning or error while running the new simulations. We hope this approach improves and facilitates the replication of our results.

Additionally, we performed another calculation to both estimate potential type 1 error and show that the sampling structure (absence of data during winter) is not driving our results. Using a permutation approach, we simulated data sets with permuted (randomly assigned) days of the year. We included an explanation of this simulation in the methods (lines 371-373). If the sampling structure influenced our results, we would expect that a large percentage of simulations would provide significant seasonal effects despite the randomization of the days of the year. Over 1000 simulations, 67 had a significant seasonal effect (6.7%) which is a bit higher than the 5% expected, but still reasonable. The new p-value threshold value to get a 5% type 1 error is 0.0416, which is much higher than the reported p-value from the data analysis. We can confidently say that the sampling structure is not driving the results.

The combination of both simulations shows that a linear seasonal effect would be captured by age and leading to a flat (non-significant) seasonal effect and that the sampling structure is not the cause of the results.

The results for both simulations were added in lines 118-130: "From the 1000 GAMMs fitted to data simulated with a seasonal effect, 75.9% found a significant effect of seasons, indicating high power to detect a seasonal effect given the simulated parameters and our data structure. From the 1000 GAMMs fitted to data simulated with a constant linear age effect, 6.2% had a significant season effect, indicating a slightly higher type 1 error than expected (5%). Based on this result from the simulations with no seasonal effect, we calculated a new critical value for the probability that respects the 5% type 1 error rate by estimating the 0.05 quantile of the p-value distribution from a null model. The 0.05 quantile was 0.0399, which can be taken as the critical value with which to estimate the significance of a seasonal effect. In our second approach, we simulated a 1000 data sets with permuted (randomly assigned) days of the year, and observed a significant seasonal effect for 6.7% of the GAMMs. The adjusted p-value threshold based on this result was 0.0416. The p-value for seasonal effects on the marmot data is 0.002 (Table 1) and therefore is considered significant. From both approaches, we concluded that our results were neither driven by our sampling nor by statistical artefact."

Given these concerns, the authors should consider other ways to use their dataset to get at the hibernation-aging hypothesis given their sampling constraints. One alternative analysis would be to focus on the sets of samples closest to the start and end of hibernation. The authors' hypothesis predicts that partial epigenetic states directly after the end hibernation should be lowest (as their chronological age has continued to increase while their epigenetic state has been under hibernation-induced stasis). As a result, comparisons of partial epigenetic states just prior to entering hibernation should be significantly higher, on average, than those for animals just emerging (in the subsequent year). Additionally, repeated measures within the same individuals could reveal how an individual's epigenetic trajectory changes longitudinally across seasons (also, please clarify how many repeated samples were available for individuals in the data set).

Response: We used 149 samples from 73 individuals (1 to 8 samples per individual). Detailed information per individual is available on the file "SampleSheet_Pinho_etal2021.csv", included in the supplementary material.

Since this study has been performed in a wild population, the trapping dates are not the same across individuals. In this regard, the partial epigenetic states before and after hibernation would not account for the different window of time between sampling dates across individuals. However, we have 11 cases of individuals with samples collected in consecutive years, where two or more samples have been collected in one year and at least one other sample was collected in the other year. These 11 cases are highlighted in the figure below:

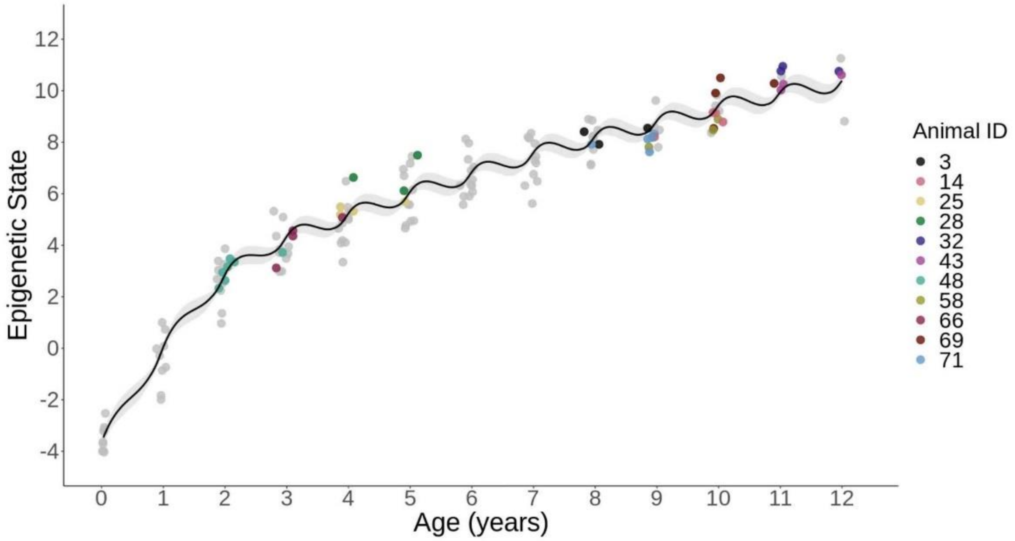

This is the same Figure 2A provided in the manuscript, with the samples used for this additional analysis in color. For a better visualization, the plots below focus on the samples from young and older individuals:

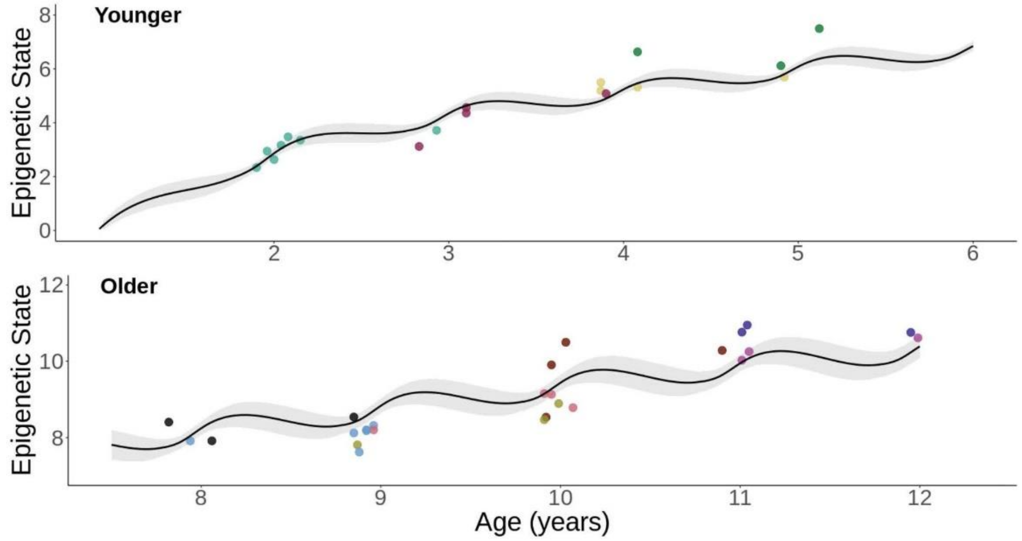

From this subset, we were able to estimate the rate of epigenetic aging per individual during the summer and winter. To measure the summer epigenetic aging rate, we subtracted the epigenetic age from the latest sample by the earliest in the active season, and divided by the number of days in

between the sampling dates. To measure the winter epigenetic aging rate, we subtracted the epigenetic age of the last sample before hibernation from the epigenetic age from the first sample collected after hibernation, divided by the number of days between the collection events. The box plot with the absolute epigenetic aging rate per day is shown below:

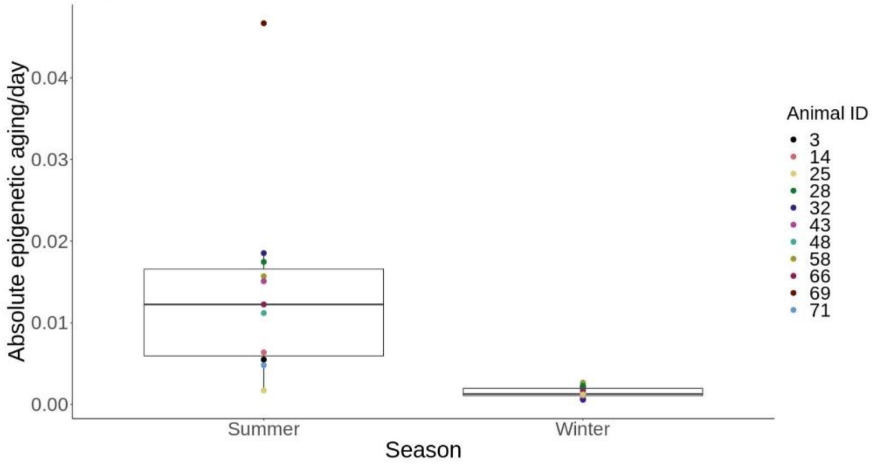

The Kruskal-Wallis rank sum test between the absolute epigenetic aging rate of summer and winter is significant (chi-squared = 13.765, df = 1, p-value = 0.0002072).

With the same samples, we also analyzed the average change in methylation levels across CpG sites during summer and winter. First, we used the 309 CpGs inputted in the EPM model. The average change in methylation levels across these sites was higher during the summer than winter (Kruskal-Wallis chi-squared = 15.783, df = 1, p-value = $7.105e^{-05}$), as seen below:

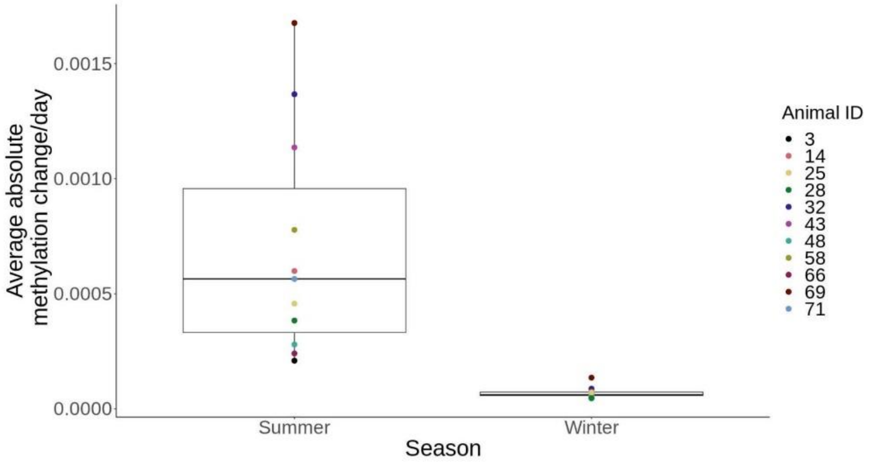

The average change in methylation levels across all sites from the mammalian array that mapped uniquely to CpG sites (and their respective flanking regions) in the yellow-bellied marmot assembly (31,388 CpGs) show a very similar pattern (Kruskal-Wallis chi-squared = 15.783, df = 1, p-value = $7.105e^{-05}$).

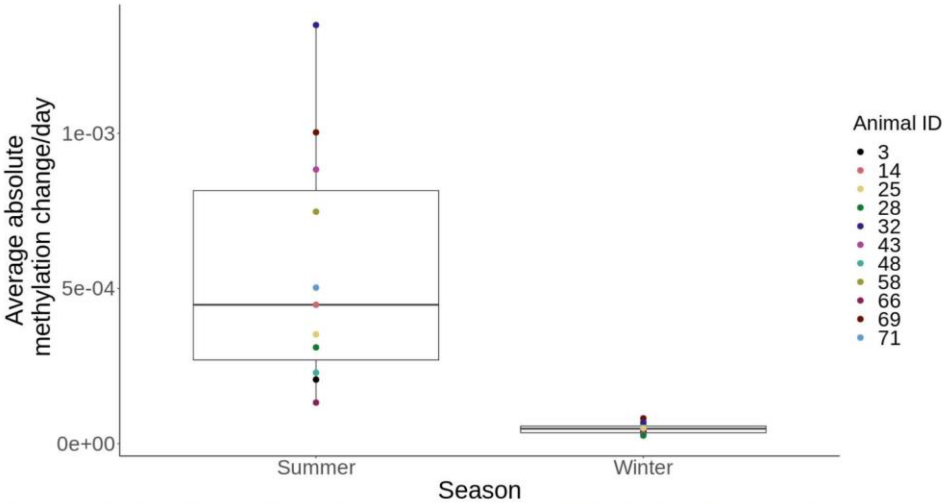

All the results from these additional analyses, focusing on individuals with samples from consecutive years, and from the simulations, reinforce the main result in the manuscript that epigenetic aging is significantly slower during hibernation than during the active season. We added these additional analyses in Appendix A and mentioned them in the main text as follows (lines 107-109): " A faster epigenetic ageing rate during the summer is also observed in the subset from our data that included only females with samples collected in consecutive years".

Finally, it would be helpful to see direct, quantitative comparisons that show that more complex nonlinear/spline fits actually significantly improve over simple linear fits (e.g., through comparing generalization error in predicting out-of-sample, formal model comparison, etc.). These should be applied both to the overall epigenetic clock/EPM analyses and to the site-by-site analyses. If all three of these analyses continue to support the manuscript's claims, the results would be much more convincing.

Response: Thank you for this suggestion. We have incorporated it and agree it has made our results more convincing (lines 342-351): " We performed model selection based on Akaike information criterion (AIC) for Generalized Additive Mixed Models (GAMMs) fitted to the EPM- or the EC-estimated epigenetic age data sets. For each epigenetic age data set, eight GAMMs with different fixed effects were compared: (1) chronological age and day of year; (2) the interaction between chronological age and day of year; (3) cubic spline function for chronological age and day of year; (4) chronological age and a cubic spline function for day of year; (5) chronological age and a cyclic cubic spline function for day of year; (6) cubic spline function for chronological age and a cubic spline function for day of year; (7) cubic spline function for chronological age and a cyclic cubic spline function for day of year; (8) the interaction (using tensor product interaction) between a cubic spline function for chronological age and a cyclic cubic spline function for day of year."

The new results are (lines 92-104): “The GAMM that best explained epigenetic ageing calculated from both EPM and EC (delta AIC > 2) had the fixed effects of chronological age (cubic spline function) and day of year (cyclic cubic spline function); and random effect of individual identity. Day of year ranged from 1 to 365, with 1 representing 1 May and 365 representing 30 April. Details on the model selection results can be found in Supplementary Table S4. For the EC epigenetic age, this model explained 96.5% of the variation (Adj. R^2) and had a residual variance of 0.362. The random effect of individual identity had no intercept variance. The age spline was significant (edf = 3.19, F = 1278, $p < 0.0001$) and the cyclic spline for day of year was not significant ($p = 1$). Details of this model are described in Supplementary Table S5.
The GAMM fitted to the EPM epigenetic state data explained 95.5% of the variation and had a residual variance of 0.294. Both smooth terms significantly influenced marmot epigenetic state ($p < 0.005$, Table 1)”

We used the model that best explained the data in the simulations (mentioned above) and in the analysis per site. In the analysis per site we wished to identify the sites that contributed the most to the pattern between epigenetic aging and season identified by the GAMM. Since we were not interested in describing the best model for each site, we preferred to keep the analysis per site simple.

Finally, the authors perform gene set enrichment for effects of day of year/seasonality. They report a number of significant gene sets, which is surprising given the small number of significantly associated CpG cites (n=126). I am concerned that these results stem from poorly calibrated tests where only a small number of sites are even associated with a given functional term. I suggest removing terms with small numbers of tested sites (e.g., less than 10 or 20) from this analysis. The MHT could also be minimized by pruning out gene sets of uninterpretable functon(e.g., “HP:0002714: Downturned corners of mouth”); currently, over 60000 different gene sets are tested, as they are unlikely to provide any real biological insight.

Response: In Figure 3C we show the CpGs with a significant effect of season from the GAMs per site, and the genes proximal to some of these top CpGs are described in the results. These are not part of the enrichment results and we added clarification in the results (lines 162-166) “Some of the most significant CpGs for both season and age effects in the GAMs are proximal to *FILIP1* exon, *ARHGEF12* intron, *ZNF521* intron, *JARID2* exon, and *AHDC1* intron (Figure 3C). The most significant CpGs for the season effect alone are proximal to *AHDC1* intron, *MAZ* exon, *CTNNA1* exon, *AUTS2* intron, and *EFNA5* exon (Figure 3C)”; and clarified it in the legend of Figure 3 “For some of the most significant CpGs, the symbol of proximal genes are provided.” (line 709-710)

However, we agree we should have been more strict with the results from the enrichment because of the small number of sites. By removing the terms with a small number of tested sites, we no longer found significant results. We removed the previous results, their discussion, and added “ and no biological function was considered significant” in line 172-173.

Minor comments:

Lines 85: What quality control was performed to exclude the 11 samples?

Response: This information has been added to the manuscript on lines 300-309: “We used two different unsupervised hierarchical clustering procedures to identify technical outliers. The first clustering procedure was based on imputed SNPs. Toward this end, we used MethylToSNP (Labarre et al., 2019) to identify CpG sites that corresponded to SNPs. The SNP data was used for unsupervised

hierarchical clustering based on Euclidean distances. Branches (clusters) of the cluster tree corresponded to multiple samples from the same animal. This allowed us to identify a small platemap error probably caused by human pipetting error. To err on the side of caution we removed these putative platemap errors from the data set. Second, we carried out average linkage hierarchical clustering based on the inter-array correlation to identify technical outliers due to an insufficient amount of DNA. The DNAm profiling from 149 samples passed quality control. These samples were collected from 73 females (1 to 8 samples per individual) with ages varying from 0.01 to 12.04 years."

Lines 226: Why was this significance threshold chosen?

Response: We added the explanation in lines 134-142: "Significance thresholds were set to $1x10^{-5}$ due to a compromise between the Bonferroni correction and the Benjamini–Hochberg FDR. The Bonferroni threshold from our data was $1.6e^{-06}$ and selected 5,317 sites from the GAM (age effect) and 5,440 from the EWAS. The Benjamini–Hochberg FDR is a less conservative false detection rate correction and selected 14,117 sites from the GAM (age effect) and 13,575 from the EWAS. The threshold used is strict particularly because methylation levels are highly correlated among CpGs, and thus the analyses per site are not independent. Haghani et al. (2021) identified 55 co-methylation modules in the Mammalian array, which would lead to a threshold of $p < 0.001$ with a Bonferroni correction"

Lines 296-297: Non-linear relationships between age and DNA methylation might be a product of technical effects (e.g. noise in methylation estimates, small sample size) or a true non-linear, biological relationship. The fact that 66% of age-associated sites had an EDF>2 could be consistent with either, but is not necessarily definitive effective of non-linear aging (especially since DNA methylation levels are bounded at 0 and 1). I'd suggest that the authors pull back on this conclusion throughout the manuscript.

Response: We agree that non-linear effects can come from either technical effects or true-non-linear effects. As mentioned above, it is unlikely that the cubic spline function forced a spurious non-linear relationship between chronological age and methylation levels. We suggest that 149 samples is sufficiently large to detect this relationship. Although not a definitive result, the observation about the EDF values from the GAMs per site is an interesting pattern that complements our results from the changes in epigenetic aging rate in different life stages. To address for the reviewer's concern, we toned down the discussion of this result by rewording the results and discussion where needed. Lines 144-146: "The edf measures the complexity of the curve, and these results suggest that many CpG sites have a non-linear relationship with chronological age (examples in Supplementary Figure S2)" and deleted its mention in the discussion (lines 201-202).

Reviewer #3 (Remarks to the Author):

Pinhoa et al., have developed an epigenetic clock on yellow-bellied marmots. The authors have also developed an epigenetic pacemaker to assist in explaining the increase longevity in animals that hibernate. The work presented provides an interesting case study for epigenetic clocks and animals that hibernate, which has not been previously explored. As the authors discuss in the manuscript, epigenetic clocks may not be able to detect or capture seasonal variations due to the penalised regression. This may have implications in other species that experience cyclic patterns other than hibernation. Although, this study is well detailed and presented, I do have some minor suggestions and comments that the authors may be able to address.

Comments

Line 240. The authors report polycomb complex targets were proximal to the CpG sites and this pattern is consistent with other mammals. The authors reference the manuscript on bioRxiv.org titled 'Universal DNA methylation age across mammalian tissues.' This study referenced and the one presented both use the Illumina chip HorvathMammalMethylChip40. This array is a useful tool to develop epigenetic clocks for mammals as it can be applied to broad range of species.
My concern is that due to the selection of CpG sites in the Illumina array, the sites used for an epigenetic clock under this system will always be enriched for polycomb complex targets and pathways relating to development and cell differentiation. If the authors used a non-targeted approach to selecting CpG sites such as reduced representation bisulphite sequencing, such as the method used in mice, would the CpG sites still be enriched for polycomb complex targets?
Mouse DNA methylation age study: https://pubmed.ncbi.nlm.nih.gov/28399939/

Response: Thank you for raising this point. In our method paper on HorvathMammalMethylChip40 (Arneson et al., 2021), we performed an enrichment for all probes on the array. The probe selection for Mammalian methylation array was based on sequence conservation and not selective for specific genes, regions, or biological processes. Based on human ChipSeq experiments, approximately 4000 probes (~11%) in the mammalian array are located in PRC2 complex binding sites. The remaining probes covers almost all other chromatin states, as discussed in Arneson et al (2021). However, we acknowledge that the array may have some bias toward PRC2 complex CpGs due to a higher sequence conservation in these regions. Unfortunately the use of RRBS data would not alleviate this valid concern since they suffer from a similar bias resulting from its focus on CpG rich regions.

Importantly, all CpGs in the mammalian array that mapped to conserved genes between marmots and humans were used as background set in our GREAT enrichment analysis. As described in lines 394-396, "GREAT tests the observed distribution of CpG neighboring genes against the expected number of sites associated with each pathway due to their representation in the mammalian array (background set)", it removes any bias resulting from the mammalian array platform.

Minor comments:
Line 123. Were sites with a negative correlation also included?

Response: Yes, we used absolute Pearson coefficients. We added "absolute" at this line and at other parts of the text that we mention "*r*" but actually mean "absolute *r*". Thank you.

Line 127. As a suggestion, 1-2 sentences of why a Generalized Additive Mixed Model was used may help readers who are not familiar with the method.

Response: We added: "Because the effects of season on epigenetic aging may be non-linear and potentially cyclical, we used Generalized Additive Mixed Models (GAMMs) to test the hibernation-aging hypothesis." (lines 90-91)

Line 180. Figure 1 shows an impressive epigenetic clock. Can the authors also provide the median absolute error rate and relative error?

Response: Added in lines 87-89, as follows: "The EC model had a median absolute error (MdAE) of 0.381 and a median relative absolute error (MdRAE) of 0.084, while the EPM model had a MdAE of 0.899 and a MdRAE of 0.145."

Line 239. 'Most CpGs in promoter regions were hypermethylated with age' – can the specific number be provided?

Response: We added this information and edited the paragraph to increase clarity (lines 151-155): "The significant CpGs were located in both genic and intergenic regions relative to transcriptional start sites (Figure S3). Compared to the background, we observed a higher proportion located at promoter regions, where most CpGs were hypermethylated with age (Figure S3). From the 3,914 CpGs used in the enrichment analysis, 396 were located in promoter regions and 87% of those were hypermethylated with age."

Even more minor:
1. Should the gene names in the manuscript be italicised?

Response: Modified; thanks!

References

Arneson, A., Haghani, A., Thompson, M. J., Pellegrini, M., Bin, S., Vu, H., Li, C. Z., Lu, A. T., Barnes, B., Hansen, K. D., Zhou, W., Breeze, C. E., Ernst, J., & Horvath, S. (2021). A mammalian methylation array for profiling methylation levels at conserved sequences. *BioRxiv*, doi: 10.1101/2021.01.07.425637. https://doi.org/https://doi.org/10.1101/2021.01.07.425637

Bieber, C., Turbill, C., & Ruf, T. (2018). Effects of aging on timing of hibernation and reproduction. *Scientific Reports*, *8*, 13881. https://doi.org/10.1038/s41598-018-32311-7

Haghani, A., Lu, A. T. T., Li, C. Z. Z., Robeck, T. R. R., Belov, K., Breeze, C. E. E., Brooke, R. T. T., Clarke, S., Faulkes, C. G. G., Fei, Z., Ferguson, S. H. H., Finno, C. J. J., Gladyshev, V. N. N., Gorbunova, V., Goya, R. G. G., Hogan, A. N. N., Hogg, C. J. J., Hore, T. A. A., Kiaris, H., … Horvath, S. (2021). DNA Methylation Networks Underlying Mammalian Traits. *BioRxiv*, 2021.03.16.435708. https://doi.org/10.1101/2021.03.16.435708

Labarre, B. A., Goncearenco, A., Petrykowska, H. M., Jaratlerdsiri, W., Bornman, M. S. R., Hayes, V. M., & Elnitski, L. (2019). MethylToSNP: Identifying SNPs in Illumina DNA methylation array data. *Epigenetics and Chromatin*, *12*, 79. https://doi.org/10.1186/s13072-019-0321-6

Decision Letter, first revision:

15th October 2021

Dear Steve,

Thank you for submitting your revised manuscript "Hibernation slows epigenetic ageing in yellow-bellied marmots" (NATECOLEVOL-210513678A). It has now been seen again by the original reviewers and their comments are below. The reviewers find that the paper has improved in revision, and therefore we'll be happy in principle to publish it in Nature Ecology & Evolution, pending minor revisions to satisfy the reviewers' final requests and to comply with our editorial and formatting guidelines.

I should stress that the remaining points of reviewer #2 must be addressed in the manuscript, including discussion of any necessary caveats.

We are now performing detailed checks on your paper and will send you a checklist detailing our editorial and formatting requirements in about a week. Please do not upload the final materials and make any revisions until you receive this additional information from us.

Thank you again for your interest in Nature Ecology & Evolution. Please do not hesitate to contact me if you have any questions.

[REDACTED]

Reviewer #1 (Remarks to the Author):

My major concern has been addressed.

Reviewer #2 (Remarks to the Author):

In this paper, Pinho et al. use an epigenetic clock and epigenetic pacemaker to investigate the effects of hibernation on aging in a well-studied population of wild marmots. This is the second time I've seen this manuscript, and several of my previous concerns about methodological artifacts have been addressed by the revisions. I have a few remaining questions and suggestions.

1) The revised simulation code now runs smoothly, making it possible to recreate the false positive rate estimates reported in the manuscript. I was surprised to see, however, that the permutation analysis produces a distribution of p-values under the null that is very enriched for p = 1 (as opposed to the typical expectation of uniformity or near-uniformity under the null). For example, re-running the code typically produces a distribution where more than 50% of p-values = 1.

I am not sure what's happening here (e.g., is there a problem with model convergence issues?), but it suggests that (i) the permutation procedure is not generating a reasonable estimate of the null; or (ii)

the test statistics/p-values are poorly calibrated.

2) It was helpful to see the new analyses comparing absolute epigenetic aging per day in summer and winter months, for the subset of individuals with longitudinal samples. This simple result is an important complement to the GAMMs in the main text, and should be included in it the main manuscript instead of the Appendix. However, rates should be calculated as epigenetic aging/day as opposed to absolute epigenetic aging/day, since the meaning of epigenetic age that drops between sequential samples (negative aging/day) is very different from the meaning of epigenetic age that increases (positive aging/day), with respect to the hibernation-aging hypothesis.

Finally, I was surprised to see that seasonal effects on average rate of change in DNA methylation for the 309 sites in the EPM model look near-identical to seasonal effects on average rates of change for the background set of 31,388 sites (plots in the response to reviewers). Under the hibernation-aging hypothesis, I expect seasonal effects on epigenetic aging/epigenetic state-related sites to look larger/distinct from all tested sites, since most sites are not age or hibernation-correlated based on both the EWAS results here and findings elsewhere. It would be helpful to present these results with the main findings and provide an explanation for why epigenetic age-related sites don't stand out (or explain why this is not expected based on the motivating hypothesis). As currently presented, the similarity with the average of ~31k sites presents a concern about possible systematic biases in the full data set that generate the winter/summer contrast.

Minor comments:
-Please give the units for the median absolute/median relative absolute error results (I think they are in years?).

Reviewer #3 (Remarks to the Author):

The authors have made improvements and in my opinion have satisfied the reviewer's comments.

Our ref: NATECOLEVOL-210513678A

21st October 2021

Dear Dr. Horvath,

Thank you for your patience as we've prepared the guidelines for final submission of your Nature Ecology & Evolution manuscript, "Hibernation slows epigenetic ageing in yellow-bellied marmots" (NATECOLEVOL-210513678A). Please carefully follow the step-by-step instructions provided in the

attached file, and add a response in each row of the table to indicate the changes that you have made. Please also check and comment on any additional marked-up edits we have proposed within the text. Ensuring that each point is addressed will help to ensure that your revised manuscript can be swiftly handed over to our production team.

We would like to start working on your revised paper, with all of the requested files and forms, as soon as possible (preferably within two weeks). Please get in contact with us immediately if you anticipate it taking more than two weeks to submit these revised files.

When you upload your final materials, please include a point-by-point response to any remaining reviewer comments.

If you have not done so already, please alert us to any related manuscripts from your group that are under consideration or in press at other journals, or are being written up for submission to other journals (see: https://www.nature.com/nature-research/editorial-policies/plagiarism#policy-on-duplicate-publication for details).

In recognition of the time and expertise our reviewers provide to Nature Ecology & Evolution's editorial process, we would like to formally acknowledge their contribution to the external peer review of your manuscript entitled "Hibernation slows epigenetic ageing in yellow-bellied marmots". For those reviewers who give their assent, we will be publishing their names alongside the published article.

Nature Ecology & Evolution offers a Transparent Peer Review option for new original research manuscripts submitted after December 1st, 2019. As part of this initiative, we encourage our authors to support increased transparency into the peer review process by agreeing to have the reviewer comments, author rebuttal letters, and editorial decision letters published as a Supplementary item. When you submit your final files please clearly state in your cover letter whether or not you would like to participate in this initiative. Please note that failure to state your preference will result in delays in accepting your manuscript for publication.

Cover suggestions

As you prepare your final files we encourage you to consider whether you have any images or illustrations that may be appropriate for use on the cover of Nature Ecology & Evolution.

Covers should be both aesthetically appealing and scientifically relevant, and should be supplied at the best quality available. Due to the prominence of these images, we do not generally select images featuring faces, children, text, graphs, schematic drawings, or collages on our covers.

We accept TIFF, JPEG, PNG or PSD file formats (a layered PSD file would be ideal), and the image should be at least 300ppi resolution (preferably 600-1200 ppi), in CMYK colour mode.

If your image is selected, we may also use it on the journal website as a banner image, and may need to make artistic alterations to fit our journal style.

Please submit your suggestions, clearly labeled, along with your final files. We'll be in touch if more information is needed.

Nature Ecology & Evolution has now transitioned to a unified Rights Collection system which will allow our Author Services team to quickly and easily collect the rights and permissions required to publish your work. Approximately 10 days after your paper is formally accepted, you will receive an email in providing you with a link to complete the grant of rights. If your paper is eligible for Open Access, our Author Services team will also be in touch regarding any additional information that may be required to arrange payment for your article.

Please note that <i>Nature Ecology & Evolution</i> is a Transformative Journal (TJ). Authors may publish their research with us through the traditional subscription access route or make their paper immediately open access through payment of an article-processing charge (APC). Authors will not be required to make a final decision about access to their article until it has been accepted. Find out more about Transformative Journals

<B>Authors may need to take specific actions to achieve compliance with funder and institutional open access mandates. For submissions from January 2021, if your research is supported by a funder that requires immediate open access (e.g. according to Plan S principles) then you should select the gold OA route, and we will direct you to the compliant route where possible. For authors selecting the subscription publication route our standard licensing terms will need to be accepted, including our self-archiving policies. Those standard licensing terms will supersede any other terms that the author or any third party may assert apply to any version of the manuscript.

Please note that you will not receive your proofs until the publishing agreement has been received through our system.

For information regarding our different publishing models please see our Transformative Journals page. If you have any questions about costs, Open Access requirements, or our legal forms, please contact ASJournals@springernature.com.

Please use the following link for uploading these materials:
[REDACTED]

If you have any further questions, please feel free to contact me.

[REDACTED]

Reviewer #1:
Remarks to the Author:

My major concern has been addressed.

Reviewer #2:
Remarks to the Author:
In this paper, Pinho et al. use an epigenetic clock and epigenetic pacemaker to investigate the effects of hibernation on aging in a well-studied population of wild marmots. This is the second time I've seen this manuscript, and several of my previous concerns about methodological artifacts have been addressed by the revisions. I have a few remaining questions and suggestions.

1) The revised simulation code now runs smoothly, making it possible to recreate the false positive rate estimates reported in the manuscript. I was surprised to see, however, that the permutation analysis produces a distribution of p-values under the null that is very enriched for p = 1 (as opposed to the typical expectation of uniformity or near-uniformity under the null). For example, re-running the code typically produces a distribution where more than 50% of p-values = 1.

I am not sure what's happening here (e.g., is there a problem with model convergence issues?), but it suggests that (i) the permutation procedure is not generating a reasonable estimate of the null; or (ii) the test statistics/p-values are poorly calibrated.

2) It was helpful to see the new analyses comparing absolute epigenetic aging per day in summer and winter months, for the subset of individuals with longitudinal samples. This simple result is an important complement to the GAMMs in the main text, and should be included in it the main manuscript instead of the Appendix. However, rates should be calculated as epigenetic aging/day as opposed to absolute epigenetic aging/day, since the meaning of epigenetic age that drops between sequential samples (negative aging/day) is very different from the meaning of epigenetic age that increases (positive aging/day), with respect to the hibernation-aging hypothesis.

Finally, I was surprised to see that seasonal effects on average rate of change in DNA methylation for the 309 sites in the EPM model look near-identical to seasonal effects on average rates of change for the background set of 31,388 sites (plots in the response to reviewers). Under the hibernation-aging hypothesis, I expect seasonal effects on epigenetic aging/epigenetic state-related sites to look larger/distinct from all tested sites, since most sites are not age or hibernation-correlated based on both the EWAS results here and findings elsewhere. It would be helpful to present these results with the main findings and provide an explanation for why epigenetic age-related sites don't stand out (or explain why this is not expected based on the motivating hypothesis). As currently presented, the similarity with the average of ~31k sites presents a concern about possible systematic biases in the full data set that generate the winter/summer contrast.

Minor comments:
-Please give the units for the median absolute/median relative absolute error results (I think they are in years?).

Reviewer #3:

Remarks to the Author:
The authors have made improvements and in my opinion have satisfied the reviewer's comments.

Author Rebuttal, first revision:

Reviewer #1:
Remarks to the Author:
My major concern has been addressed.

Response: We are grateful for your feedback and that you are now happy with the revised manuscript.

Reviewer #2:
Remarks to the Author:
In this paper, Pinho et al. use an epigenetic clock and epigenetic pacemaker to investigate the effects of hibernation on aging in a well-studied population of wild marmots. This is the second time I've seen this manuscript, and several of my previous concerns about methodological artifacts have been addressed by the revisions. I have a few remaining questions and suggestions.

1) The revised simulation code now runs smoothly, making it possible to recreate the false positive rate estimates reported in the manuscript. I was surprised to see, however, that the permutation analysis produces a distribution of p-values under the null that is very enriched for p = 1 (as opposed to the typical expectation of uniformity or near-uniformity under the null). For example, re-running the code typically produces a distribution where more than 50% of p-values = 1.

I am not sure what's happening here (e.g., is there a problem with model convergence issues?), but it suggests that (i) the permutation procedure is not generating a reasonable estimate of the null; or (ii) the test statistics/p-values are poorly calibrated.

Response: We are glad that the code runs smoothly on other computers now. Looking closely at the distribution of p-values for the permutation, we see more than 50% of simulations with a p-value > 0.9. As mentioned by reviewer 2, in most cases, we would expect a near uniform distribution of p-values with the permutation. Given the histogram with 10 bins (plot below on the left), we should expect each bin to contain about 100 simulated results. This problem mainly arises from using cyclical splines (the distribution of p-values with the permutation for the age effect is uniform). The cyclical spline has an additional constraint of joining the start and the end of the spline (i.e., there is no discontinuity). This added constraint leads to an additional penalty which potentially shrinks the null space of the model and the associated smoothing parameter. Therefore, with cyclic effects of small sizes, the model might struggle to adequately estimate the cyclic spline and instead penalize it strongly. This is illustrated by the large number of simulations with an edf (estimated degree of freedom) close to zero (plot below, on the right). The edf is expected to be 1 (linear) or above (non-linear). An edf lower than 1 is symptomatic of

shrinkage of the null space. An edf of zero (or close to zero) indicates that the term is not informative in the model and does not explain any variation in the data, similarly to an elastic-net penalty. So, with cyclical splines under the null, the GAMM might estimate an edf close to zero and p-value close to 1. However, if it does not (because the effect size is large enough), the edf and associated probability are estimated adequately. Because of this, when simulating under the null, we should expect that simulations with small effects will be either estimated adequately (p-value < 0.4) or penalized out (p-value > 0.9). In this regard, the distribution of the p-value obtained from the permutation on the seasonal effect is the expected distribution according to the GAMM model specification (uniform distribution of p-values from 0 to 0.3-0.4 and a peak at 1). This does not affect the validity of our results since we estimated the type I error from the proportion of resampling with a p-value under 0.05, for which there is no estimation bias.

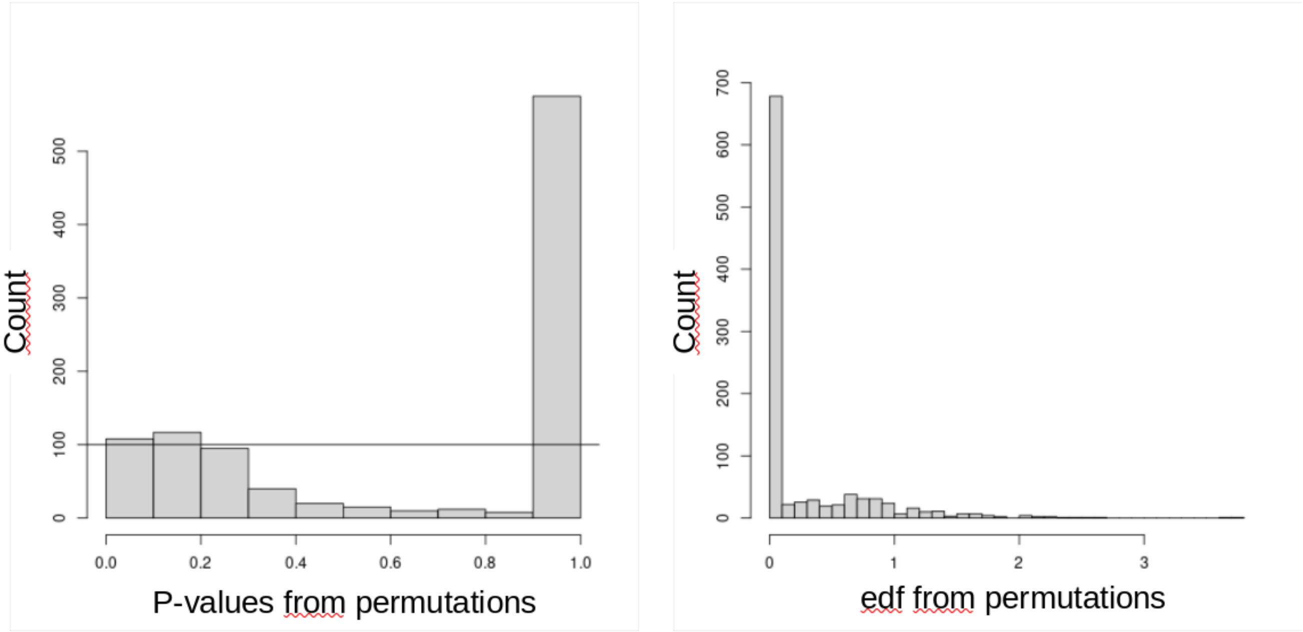

2) It was helpful to see the new analyses comparing absolute epigenetic aging per day in summer and winter months, for the subset of individuals with longitudinal samples. This simple result is an important complement to the GAMMs in the main text, and should be included in it the main manuscript instead of the Appendix. However, rates should be calculated as epigenetic aging/day as opposed to absolute epigenetic aging/day, since the meaning of epigenetic age that drops between sequential samples (negative aging/day) is very different from the meaning of epigenetic age that increases (positive aging/day), with respect to the hibernation-aging hypothesis.

Response: Thank you for this suggestion. We had put the absolute values of epigenetic ageing rate because we were more interested in the amount of change than the direction of the change. Our hypothesis suggests that the amount of change during hibernation would be minimal when compared to the active season. The plot using epigenetic ageing/day is not too different from the plot with absolute values because most individuals have increased rates in the active season. Only 2 individuals have negative rates during the active season. We agree that the epigenetic ageing/day plot is more informative and this plot has been added in the main text (lines 437-441) and we refer to it throughout the manuscript (Results: lines 101-105; Method: lines 359-368).

Finally, I was surprised to see that seasonal effects on average rate of change in DNA methylation for the 309 sites in the EPM model look near-identical to seasonal effects on average rates of change for the background set of 31,388 sites (plots in the response to reviewers). Under the hibernation-aging hypothesis, I expect seasonal effects on epigenetic aging/epigenetic state-related sites to look larger/distinct from all tested sites, since most sites are not age or hibernation-correlated based on both the EWAS results here and findings elsewhere. It would be helpful to present these results with the main findings and provide an explanation for why epigenetic age-related sites don't stand out (or explain why this is not expected based on the motivating hypothesis). As currently presented, the similarity with the average of ~31k sites presents a concern about possible systematic biases in the full data set that generate the winter/summer contrast.

Response: It is important to notice that the hibernation-ageing hypothesis does not require that age-unrelated CpGs are unaffected by hibernation. Hibernation substantially modifies several aspects of individual biology and metabolism, therefore changes at the epigenome are expected. The influence of hibernation on epigenetic ageing is evident because CpGs with high chronological-age predictive power show slower change rates during hibernation, and the influence of seasons on other biological processes may be equally possible. Horvath (2013) proposed that the epigenetic clock is a result of the cumulative effects of an epigenetic maintenance system (EMS). Such machinery would impact the epigenome as a whole and, assuming the EMS hypothesis holds, our results suggest that this machinery is suppressed in hibernating individuals.

The similar results among the 309 and 31k sites are not entirely surprising because the CpGs from the Mammalian array are not completely independent. Haghani et al. (2021) identified 55 co-methylation modules in the mammalian array using an unbiased clustering of individual citosines from 176 species. From those, 31 modules could be associated with life history traits. In our dataset, the average change in methylation levels computed for 309 and 31k CpGs were different, however, the correlation between them was 0.91. Given that we have only 22 observations for this analysis and the residuals are not normal when using a parametric test, we used a non-parametric test (Kruskall wallis sum-rank test). A test on ranks (instead of true values) of highly correlated data sets is expected to have similar results.

To further explore the relationships of the CpGs with chronological age and season, we subtracted the winter methylation change rate from the summer rate for each site per individual, and measured the mean

difference in rates per site. Thus, a CpG with a high mean difference between seasons (represented by the y axis below) is highly influenced by seasonality, with a higher methylation change rate during the active season than during hibernation. These mean values were plotted against the CpGs' Pearson correlation values with chronological age, as shown below:

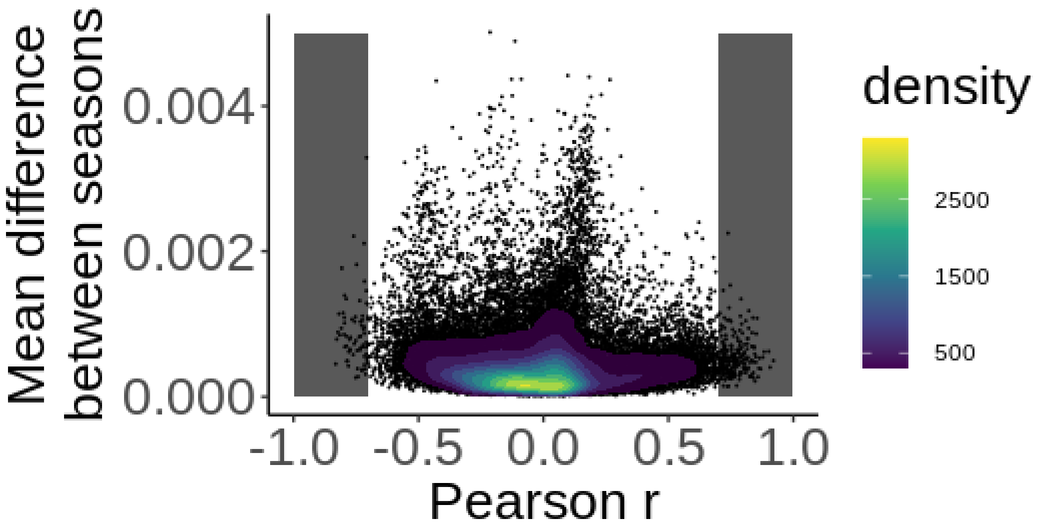

These plots cannot lead to strong conclusions because the measures per site were calculated from only 11 individuals, but a few patterns can be observed. The first plot shows that chronological age is associated with more CpGs than the 309 selected for the EPM, which are highlighted by the grey rectangles. Our cutoff for selecting sites to input in the EPM model was high (an minimum absolute *r* of 0.7), and, for example, were we to choose a threshold of 0.5, we would have developed the EPM with 2,490 sites. In Appendix A we show that, even when CpGs that were weakly associated with age (absolute r > 0.1) were inputted in the EPM, the effect of chronological age was still detected in the GAMMs. Also, the CpGs inputted in the EPM are not the sites where the mean difference in methylation rates between seasons is the highest. There are many other sites, including sites with high and low Pearson *r* values with chronological age, that seem to be affected by seasonality. This helps explain why the results from the 309 sites are similar to the results from the 31k sites, and implies an epigenome-level effect of seasons.

The plots developed with the 11 individuals with samples collected in consecutive years have an important limitation. We compared changes in methylation in between individual captures, but these methylation changes are not necessarily ordered. It is possible that the methylation values for many of the sites are not accumulating in an ordered fashion. In this regard, the EPM model estimated epigenetic states from ordered methylation changes (with chronological age), and allowed us to use a much larger number of samples. The EPM results are therefore the main contribution for testing the hibernation-ageing hypothesis and the plots and results from the subsample of these 11 individuals are added in Appendix A.

We added the new plots and the above explanation in Appendix A and are mentioning this result in line 105-109 of the main text: ”We observed similar trends for the average change in methylation levels across all CpG sites from the mammalian array, suggesting that seasons affect methylation levels at the epigenome level and may not be restricted to age-related sites (Appendix A). In this regard, hibernation may not only influence epigenetic ageing, but also other biological processes.”

Minor comments:
-Please give the units for the median absolute/median relative absolute error results (I think they are in years?).

Response: Done.

Reviewer #3:
Remarks to the Author:
The authors have made improvements and in my opinion have satisfied the reviewer's comments.

Response: We are grateful for your feedback and that you are now happy with the revised manuscript.

References

Haghani, A. *et al.* DNA Methylation Networks Underlying Mammalian Traits. Preprint at https://www.biorxiv.org/content/10.1101/2021.03.16.435708v1 (2021).

Horvath, S. DNA methylation age of human tissues and cell types. *Genome Biol.* **14,** R115 (2013).

Final Decision Letter:

20th January 2022

Dear Steve,

We are pleased to inform you that your Article entitled "Hibernation slows epigenetic ageing in yellow-bellied marmots", has now been accepted for publication in Nature Ecology & Evolution.

Please make sure to make the data available on Omnibus at the time of publication.

Over the next few weeks, your paper will be copyedited to ensure that it conforms to Nature Ecology

and Evolution style. Once your paper is typeset, you will receive an email with a link to choose the appropriate publishing options for your paper and our Author Services team will be in touch regarding any additional information that may be required.

After the grant of rights is completed, you will receive a link to your electronic proof via email with a request to make any corrections within 48 hours. If, when you receive your proof, you cannot meet this deadline, please inform us at rjsproduction@springernature.com immediately.

You will not receive your proofs until the publishing agreement has been received through our system.

Due to the importance of these deadlines, we ask you please us know now whether you will be difficult to contact over the next month. If this is the case, we ask you provide us with the contact information (email, phone and fax) of someone who will be able to check the proofs on your behalf, and who will be available to address any last-minute problems . Once your paper has been scheduled for online publication, the Nature press office will be in touch to confirm the details.

Acceptance of your manuscript is conditional on all authors' agreement with our publication policies (see www.nature.com/authors/policies/index.html). In particular your manuscript must not be published elsewhere and there must be no announcement of the work to any media outlet until the publication date (the day on which it is uploaded onto our web site).

Please note that <i>Nature Ecology & Evolution</i> is a Transformative Journal (TJ). Authors may publish their research with us through the traditional subscription access route or make their paper immediately open access through payment of an article-processing charge (APC). Authors will not be required to make a final decision about access to their article until it has been accepted. Find out more about Transformative Journals

<B>Authors may need to take specific actions to achieve compliance with funder and institutional open access mandates. For submissions from January 2021, if your research is supported by a funder that requires immediate open access (e.g. according to Plan S principles) then you should select the gold OA route, and we will direct you to the compliant route where possible. For authors selecting the subscription publication route our standard licensing terms will need to be accepted, including our self-archiving policies. Those standard licensing terms will supersede any other terms that the author or any third party may assert apply to any version of the manuscript.

In approximately 10 business days you will receive an email with a link to choose the appropriate publishing options for your paper and our Author Services team will be in touch regarding any additional information that may be required.

You will not receive your proofs until the publishing agreement has been received through our system.

If you have any questions about our publishing options, costs, Open Access requirements, or our legal forms, please contact ASJournals@springernature.com

An online order form for reprints of your paper is available at https://www.nature.com/reprints/author-reprints.html. All co-authors, authors' institutions and authors' funding agencies can order reprints using the form appropriate to their geographical region.

We welcome the submission of potential cover material (including a short caption of around 40 words) related to your manuscript; suggestions should be sent to Nature Ecology & Evolution as electronic files (the image should be 300 dpi at 210 x 297 mm in either TIFF or JPEG format). Please note that such pictures should be selected more for their aesthetic appeal than for their scientific content, and that colour images work better than black and white or grayscale images. Please do not try to design a cover with the Nature Ecology & Evolution logo etc., and please do not submit composites of images related to your work. I am sure you will understand that we cannot make any promise as to whether any of your suggestions might be selected for the cover of the journal.

You can now use a single sign-on for all your accounts, view the status of all your manuscript submissions and reviews, access usage statistics for your published articles and download a record of your refereeing activity for the Nature journals.

To assist our authors in disseminating their research to the broader community, our SharedIt initiative provides you with a unique shareable link that will allow anyone (with or without a subscription) to read the published article. Recipients of the link with a subscription will also be able to download and print the PDF.

You can generate the link yourself when you receive your article DOI by entering it here: <a href="http://authors.springernature.com/share">http://authors.springernature.com/share<a>.

[REDACTED]

P.S. Click on the following link if you would like to recommend Nature Ecology & Evolution to your librarian http://www.nature.com/subscriptions/recommend.html#forms

** Visit the Springer Nature Editorial and Publishing website at www.springernature.com/editorial-and-publishing-jobs for more information about our career opportunities. If you have any questions please click here.**
